# Expression and characterization of protein disulfide isomerase family proteins in bread wheat

**DOI:** 10.1186/s12870-015-0460-2

**Published:** 2015-03-04

**Authors:** Shizuka Kimura, Yuki Higashino, Yuki Kitao, Taro Masuda, Reiko Urade

**Affiliations:** Division of Agronomy and Horticultural Science, Graduate School of Agriculture, Kyoto University, Uji, Kyoto 611-0011 Japan

**Keywords:** Protein disulfide isomerase, Oxidative refolding, Starchy endosperm, Aleurone cell, Endoplasmic reticulum, Bread wheat

## Abstract

**Background:**

The major wheat seed proteins are storage proteins that are synthesized in the rough endoplasmic reticulum (ER) of starchy endosperm cells. Many of these proteins have intra- and intermolecular disulfide bonds. In eukaryotes, the formation of most intramolecular disulfide bonds in the ER is thought to be catalyzed by protein disulfide isomerase (PDI) family proteins. The cDNAs that encode eight groups of bread wheat (*Triticum aestivum L*.) PDI family proteins have been cloned, and their expression levels in developing wheat grains have been determined. The purpose of the present study was to characterize the enzymatic properties of the wheat PDI family proteins and clarify their expression patterns in wheat caryopses.

**Results:**

PDI family cDNAs, which are categorized into group I (*TaPDIL1Aα*, *TaPDIL1Aβ*, *TaPDIL1Aγ*, *TaPDIL1Aδ*, and *TaPDIL1B*), group II (*TaPDIL2*), group III (*TaPDIL3A*), group IV (*TaPDIL4D*), and group V (*TaPDIL5A*), were cloned. The expression levels of recombinant TaPDIL1Aα, TaPDIL1B, TaPDIL2, TaPDIL3A, TaPDIL4D, and TaPDIL5A in *Escherichia coli* were established from the cloned cDNAs. All recombinant proteins were expressed in soluble forms and purified. Aside from TaPDIL3A, the recombinant proteins exhibited oxidative refolding activity on reduced and denatured ribonuclease A. Five groups of PDI family proteins were distributed throughout wheat caryopses, and expression levels of these proteins were higher during grain filling than in the late stage of maturing. Localization of these proteins in the ER was confirmed by fluorescent immunostaining of the immature caryopses. In mature grains, the five groups of PDI family proteins remained in the aleurone cells and the protein matrix of the starchy endosperm.

**Conclusions:**

High expression of PDI family proteins during grain filling in the starchy endosperm suggest that these proteins play an important role in forming intramolecular disulfide bonds in seed storage proteins. In addition, these PDI family proteins that remain in the aleurone layers of mature grains likely assist in folding newly synthesized hydrolytic enzymes during germination.

**Electronic supplementary material:**

The online version of this article (doi:10.1186/s12870-015-0460-2) contains supplementary material, which is available to authorized users.

## Background

The major seed proteins of bread wheat (*Triticum aestivum* L.) include gliadins and glutenins. These storage proteins are synthesized in the rough endoplasmic reticulum (ER) of starchy endosperm cells and accumulate in two kinds of protein bodies derived from the ER and protein storage vacuoles [1-3]. Many of the wheat seed storage proteins have intramolecular disulfide bonds [4]. For example, gliadins have three to four intramolecular disulfide bonds in the C-terminal domain. In the case of γ-gliadins, formation of intramolecular disulfide bonds in the ER has been demonstrated to be essential for transport to the Golgi apparatus and deposition into protein bodies [5-8]. Therefore, mutations in cysteine residues or the reduction of disulfide bonds result in precipitation into insoluble aggregates in the ER [6-8].

Generally, the formation of disulfide bonds in proteins synthesized in the rough ER occurs mainly via dithiol/disulfide transfer reactions catalyzed by protein disulfide isomerase (PDI) (EC 5.3.4.1) and PDI-related proteins in eukaryotes [9]. PDI has two thioredoxin domains that contain the redox active site CGHC (**a** and **a’**) and two inactive domains (**b** and **b’**) [10]. Other PDI family members contain one or more thioredoxin domains [11]. In dicotyledonous *Arabidopsis*, a set of 22 orthologs of known PDI family proteins was discovered by a genome-wide search, and these orthologs were separated into phylogenetic groups I-X [12].

The physiological functions and biochemical properties of several plant PDI family members have been studied. AtPDIL5, an *Arabidopsis* ortholog of group I PDI family proteins, is expressed in endothelial cells of developing seeds and traffics together with the cysteine proteases RD21 and CP43 from the ER through the Golgi to vacuoles [13]. Studies of an AtPDIL5-null mutant revealed that AtPDIL5 is required for proper seed development and regulates the timing of programmed cell death by chaperoning and inhibiting cysteine proteases and serving as a redox-sensitive protease regulator during their trafficking to vacuoles before endothelial cells undergo programmed cell death. AtPDIL2-1, an *Arabidopsis* ortholog of group IV PDI family proteins, has been shown to act in maternal sporophytic tissues to affect embryo sac development [14]. A truncated AtPDIL2-1 mutant has been demonstrated to function as a gain-of-function mutant in sporophytic tissues and to affect ovule structure and impede embryo sac development, thereby disrupting pollen tube guidance.

In *Oldenlandia affinis*, a coffee family (Rubiaceae) plant, Oa PDI, an *Oldenlandia* ortholog of group I PDI family proteins, has been shown to be involved in the biosynthesis of the knotted circular proteins termed cyclotides [15]. In addition, Oa PDI dramatically enhances the oxidative folding of kalata B1 at physiological pH *in vitro*.

GmPDIL-1, GmPDIL-2, GmPDIL-3, GmPDIS-1 and −2, and GmPDIM, soybean orthologs of groups I-V PDI family proteins have been identified, and the recombinant proteins of GmPDIL-1, GmPDIL-2, GmPDIS-1, GmPDIS-2 and GmPDIM, but not GmPDIL-3, have been demonstrated to possess oxidative refolding activities [16-19]. In addition, GmPDIL-1, GmPDIL-2, GmPDIS-1, and GmPDIM have been shown to be involved in the folding of the soybean seed storage proteins proglycinin and β-conglycinin in the ER of cotyledon cells.

In monocotyledonous rice, grains of the mutant *eps*, which lack the rice ortholog (PDIL1-1) of group I PDI proteins, fail to generate the normal prolamin-containing protein bodies-I and accumulate the 57-kD proglutelin polypeptide in aggregate with the prolamin polypeptides via intermolecular disulfide bonds in small ER-derived protein bodies of uniform size (0.5 m in diameter) [20]. In addition, PDIL1-1 is asymmetrically distributed within the cortical cisternal ER, and this ortholog is essential for the maturation of proglutelin only when its rate of synthesis significantly exceeds its export from the ER. These findings suggest that rice PDIL1-1 helps retain proglutelin in the cisternal ER lumen until it attains competence for ER export [21]. Furthermore, analysis of the T-DNA insertion mutant revealed that rice PDIL1-1 deficiency causes a chalky phenotype, thick aleurone layer, lower protein content, and higher free sugar content in grains than the wild-type rice protein, suggesting that rice PDIL1-1 is involved in regulatory activities for various proteins that are essential for the synthesis of grain components [22]. Rice PDIL2;3, a rice ortholog of group V PDI family proteins, has been shown to be efficiently targeted to the surface of protein bodies-I in a redox active site-dependent manner and to play an important role in the accumulation of Cys-rich 10-kD prolamin (crP10) in the core of PB-I [23]. Complementation experiments using *eps* have indicated that rice PDIL2;3 and PDIL1;1 are not functionally redundant for disulfide bond formation in structurally diverse storage proteins and that these proteins play distinct roles in protein body development.

In wheat, the importance of PDI family proteins in seed storage protein folding and accumulation has been long predicted. Oxidative refolding activity was first reported in the embryo of bread wheat in 1978 [24], and activity in the endosperm was subsequently reported [25,26]. In 1995, a cDNA encoding a typical PDI was cloned. In addition, a 60-kD glycoprotein, which had a partial amino acid sequence homologous to the amino acid sequence predicted from the cDNA, was purified from grains of bread wheat by Shimoni *et al*. [27,28]. Since then, many cDNAs encoding wheat PDI family proteins have been identified [29-37]. Furthermore, d’Aloisio *et al*. reported the cloning of cDNAs encoding one typical PDI categorized into group I and eight PDI family proteins categorized into groups II-VIII [34]. These investigators also found that genes encoding these PDI family proteins are located in chromosome regions syntenic to those in rice [36]. Quantitative analysis of mRNAs transcribed from these genes revealed that these genes were constitutively expressed in all tissues examined but were characterized by different expression profiles [34]. Based on mRNA expression data, PDI family proteins (especially in groups I-V) have been proposed to play essential roles in grain development; however, the enzymatic activities of these individual proteins have not been characterized. In this study, we report the cloning of cDNAs encoding bread wheat PDI family proteins of groups I-V (TaPDIL1A, TaPDIL1B, TaPDIL2, TaPDIL3A, TaPDIL4D, and TaPDIL5A) and characterization of the enzymatic properties of their recombinant proteins. In addition, this report describes the expression and subcellular localization of these PDI family proteins in developing and mature caryopses.

## Results and discussion

### Cloning of wheat PDI family genes

**Figure 1:**
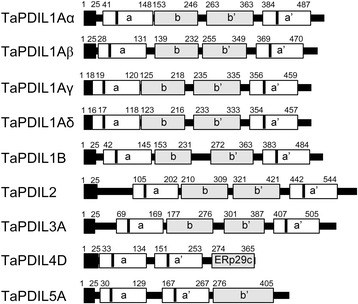
**Wheat PDI family protein domain structures deduced from the cDNA clones.** The boxes indicate the domain boundaries predicted by an NCBI conserved domain search. Black boxes in domains a and a’ represent the CXXS/C motif. The N-terminal black box represents a signal peptide that was predicted using SignalP-4.0 euk software.

**Figure 2:**
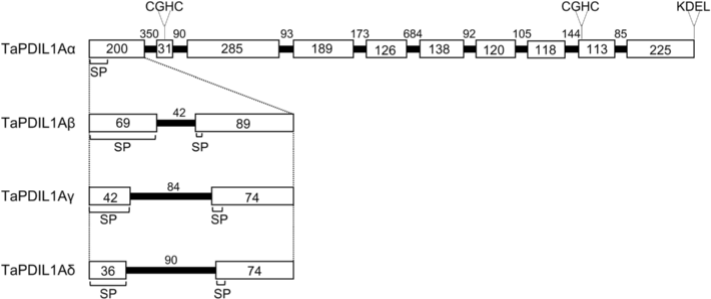
**Splicing sites in** ***TaPDIL1Aα*** **,** ***TaPDIL1Aβ*** **,** ***TaPDIL1Aγ*** **, and** ***TaPDIL1Aδ*** **.** Open boxes indicate exons, and solid black lines denote introns. The numbers indicate the size of each exon and intron (bp). The positions of the signal peptide (SP), the two CGHC motifs, and the C-terminal KDEL sequence are indicated.

In this study, we harvested mRNAs of bread wheat PDI family protein orthologs from groups I, II, III, IV, and V [12] from the immature caryopses and performed PCR using primer sets designed from their nucleotide sequences as reported previously [34]. Next, we generated and cloned the cDNAs encoding these PDI orthologs. We obtained five clones categorized into group I (*TaPDIL1Aα* [DDBJ:AB933341], *TaPDIL1Aβ* [DDBJ:AB933345], *TaPDIL1Aγ* [DDBJ:AB933342], *TaPDIL1Aδ* [DDBJ:AB933343], and *TaPDIL1B* [DDBJ:AB933344]) and one clone categorized into group II (*TaPDIL2,* [DDBJ:AB933346]), group III (*TaPDIL3A*, [DDBJ:AB933347]), group IV (*TaPDIL4D*, [DDBJ:AB933348]) and group V (*TaPDIL5A,* [DDBJ:AB933349]) (Table 1). Among these clones, *TaPDIL1Aβ*, *TaPDIL1Aγ*, *TaPDIL1Aδ*, and *TaPDIL2* have novel nucleotide sequences. The domain structures predicted from the amino acid sequences encoded by these cDNAs are shown in Figure 1. PDI family proteins categorized into group I are members of the representative eukaryotic PDI, which possesses an N-terminal signal sequence, two thioredoxin-like motifs with a CGHC active site (**a** and **a’** domains), two putative thioredoxin-folded domains without active site (**b** and **b’** domains), and a C-terminal KDEL sequence that functions as an ER retention signal [38,39]. In earlier studies [33,34], three genes (*TaPDIL1-1 [CPDI4A], TaPDIL1-2,* and *TaPDIL1-3*) encoding group I PDI family proteins were shown to be located on chromosome arms 4AL, 4BS, and 4DS, respectively. Recently, the wheat genome draft sequences were deposited in the databases of the wheat portal (http://wheat-urgi.versailles.inra.fr/Seq-Repository/) [40]. Nucleotide sequences of the cDNAs of *TaPDIL1Aα, TaPDIL1Aβ, TaPDIL1Aγ*, and *TaPDIL1Aδ* were coincident with those of the *TaPDIL1-1* gene and contigs on chromosome 4AL. Alignments of the nucleotide sequences of these cDNA with the genomic nucleotide sequence of *TaPDIL1A* [GenBank:AJ868102] suggested that they are produced by intron retention-type alternative splicing of the first exon (Figure 2) [41]. *TaPDIL1Aα*, *TaPDIL1Aβ*, *TaPDIL1Aγ*, and *TaPDIL1Aδ* cDNAs encoded proteins of 515, 501, 487, and 485 amino acids, respectively (Table 2, Additional file 1: Figure S1). TaPDIL1Aβ, TaPDIL1Aγ, and TaPDIL1Aδ lack the amino acid sequences Pro21-A34, Ala15-Leu42, and Ala13-Leu42 found in TaPDIL1Aα, respectively. The putative signal peptides of TaPDIL1Aγ and TaPDIL1Aδ (Met1-Ala18 and Met1-Ala16, respectively) were shorter than those of TaPDIL1Aα and TaPDIL1Aβ (Met1-Ala25 and Met1-Ala26, respectively). The effects of such alterations in the signal peptide on TaPDIL1A protein targeting to the ER are unclear. The nucleotide sequence of *TaPDIL1B* cDNA was matched to that of GPDIA-4B, the *TaPDIL1-2* genomic gene [GenBank: AJ868103] [33,34], and contigs on chromosome 4BS. TaPDIL1Aα, −1β, −1γ, and -1δ and TaPDIL1B have a conserved arginine involved in the regulation of the active site redox potential and a conserved glutamic acid that facilitates the release of the active site from a mixed disulfide with substrate in human PDI [42-44] in both the **a** and **a’** domains (Table 2 and Additional files 1: Figure S1 and Additional file: 2: Figure S2).

**Table 1:** **Wheat PDI family proteins cloned in this study**

**Clone**	**ORF (nt)**	**Accession number**	**Clone name reported previously**	**Accession number**
TaPDIL1Aα	1545	AB933341	CPDI4A*, TaPDIL1-1a**, TaPDIL1-1c*	AJ868105
TaPDIL1Aβ	1503	AB933345	-	-
TaPDIL1Aγ	1461	AB933342	-	-
TaPDIL1Aδ	1455	AB933343	-	-
TaPDIL1B	1536	AB933344	CPDI4B*, TaPDIL1-1b**	AJ868106
TaPDIL2	1755	AB933346	-	-
TaPDIL3A	1623	AB933347	TaPDIL3-1**	FN555317
TaPDIL4D	1101	AB933348	TaPDIL4-1**	FN555318
TaPDIL5A	1320	AB933349	TaPDIL5-1a**	FN555320

*reference 33, **reference 34.

**Table 2:** **Characteristics of the wheat PDI family proteins cloned in this study**

**Name**	**Total amino acid residues**	Signal peptide*	**Molecular weight**	**pI**	**Conserved active site**	**Consensus N-glycosylation site(s)**	**Conserved arginine(s)**	**Conserved charge pairs**	**ER retention signal**
TaPDIL1Aα	515	1–25	56579.13	4.99	C68GHC71, C412GHC415	N283	R136, R475	E62/K96, E406/K439	KDEL
TaPDIL1Aβ	501	1–26	55256.73	5.07	C54GHC57, C398GHC401	N269	R122, R461	E48/K82, E392/K425	KDEL
TaPDIL1Aγ	487	1–18	53977.23	5.10	C40GHC43, C384GHC387	N255	R108, R447	E34/K68, E378/K411	KDEL
TaPDIL1Aδ	485	1–16	53679.83	5.10	C38GHC41, C382GHC385	N253	R106, R445	E32/K66, E376/K399	KDEL
TaPDIL1B	512	1–25	56425.02	5.03	C68GHC71, C412GHC415	N283	R136, R475	E62/K96, E406/K439	KDEL
TaPDIL2	585	1–28	63588.76	4.61	C129GHC132, C470GHC473	N109, N212	R136, R535	E123/K157, E464/K497	KDEL
TaPDIL3A	541	1–23	59594.01	4.95	C96ERS99, C435VDC438	N150	R160	E429K462	KDEL
TaPDIL4D	367	1–30	40260.95	6.17	C60GHC63, C179GHC182	-	R125, R244	E54/K87, E173/K211	-
TaPDIL5A	440	1–22	47207.89	5.30	C57GHC60, C194GHC197	N164, N170	R119, R257	E51/K89, E188/K226	NDEL

*Signal peptides were predicted using SignalP 4.1 Server by the Technical University of Denmark.

The *TaPDIL2* cDNA encoded a 585- amino acid protein (Figure 1 and Table 2), and the nucleotide sequence of *TaPDIL2* cDNAs was 99% identical to that of *TaPDIL2-1* [34]. No contig sequences matched with the sequence of *TaPDIL2* cDNA found in the wheat genome draft sequences. The putative amino acid sequence of TaPDIL2 was also 99% identical to that of TaPDIL2-1 (Additional file 3: Figure S3). TaPDIL2 was categorized as a group II PDI family protein that possesses an N-terminal signal sequence, aspartic acid-rich flanking region, two thioredoxin-like motifs with a CGHC active site (**a** and **a’** domains), two putative thioredoxin-folded domains without active sites (**b** and **b’** domains), and a C-terminal KDEL sequence. TaPDIL2 also has a conserved arginine and a conserved glutamic acid in both the **a** and **a’** domains (Table 2 and Additional file 4: Figure S4).

The *TaPDIL3A* cDNA encoded a 541-amino acid protein (Table 2), and the nucleotide sequence was matched to contigs on chromosome 7AS. TaPDIL3A was categorized as a group III PDI family protein and possesses the same domain structure as TaPDIL1A (Figure 1). However, the sequences of the active sites in the **a** and **a’** domains (CERS and CVDC) differ from the representative motif CGHC. In addition, TaPDIL3A lacks the conserved glutamic acid in the **a** domain, as well as the conserved arginine in the **a** and **a’** domains (Additional file 5: Figure S5).

The nucleotide sequence of the *TaPDIL4D* cDNA was matched to that of contigs on chromosome 1AS. TaPDIL4D was categorized as a group IV PDI family protein, which is unique to plants and possesses an N-terminal signal peptide, two thioredoxin-like motifs with a CGHC active site (**a** and **a’** domains), and a domain homologous to the C-terminal domain of mammalian ERp29 (Figure 1 and Table 2) [45].

*TaPDIL5A* was categorized as a group V PDI family protein. This sequence encodes a protein of 440 amino acids that is homologous to mammalian P5 [46], and the nucleotide sequence of *TaPDIL5A* cDNA was matched to that of contigs on chromosome 5AL. TaPDIL5A possesses an N-terminal signal peptide, two thioredoxin-like motifs with a CGHC active site (**a** and **a’** domains), a putative thioredoxin-folded **b** domain, and a C-terminal NDEL sequence similar to KDEL (Figure 1 and Table 2). TaPDIL4D and TaPDIL5A contain a conserved arginine and a conserved glutamic acid in both the **a** and **a’** domains (Table 2 and Additional files 6: Figure S6 and Additional files 7: Figure S7).

### Expression and characterization of recombinant wheat PDI family proteins

**Figure 3:**
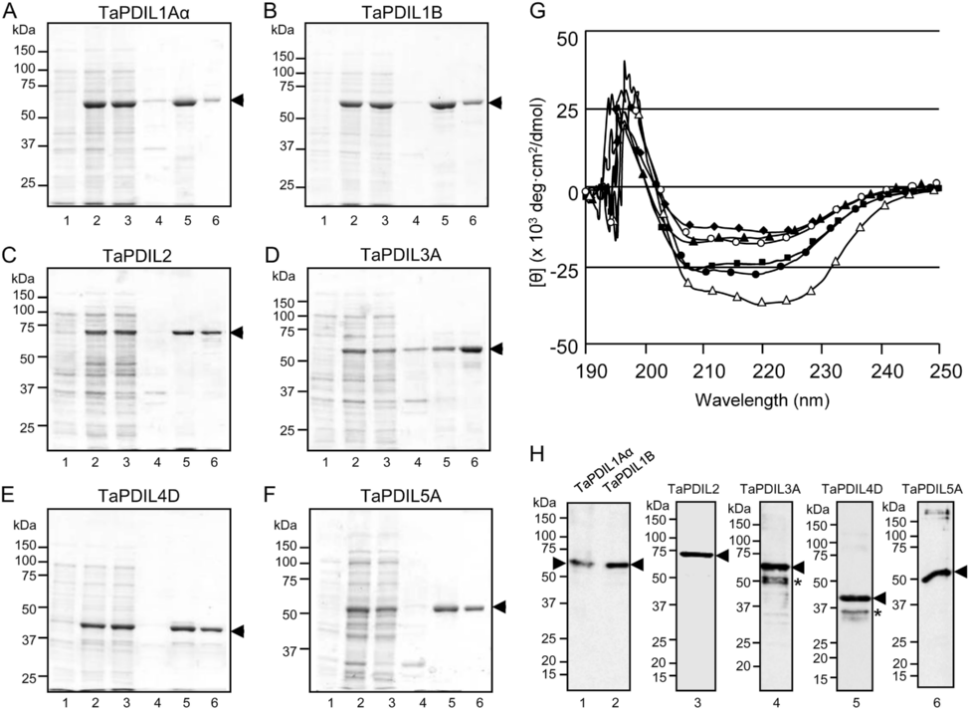
**Expression of recombinant wheat PDI family proteins.** Recombinant TaPDIL1Aα **(A)**, TaPDIL1B **(B)**, TaPDIL2 **(C)**, TaPDIL3A **(D)**, TaPDL4D **(E)**, and TaPDIL5A **(F)** were expressed in *E. coli*. cells transformed with each expression plasmid and incubated with 0.4 mM IPTG at 30°C for 0 (lane 1) or 20 h (lane 2). Soluble (lane 3) and insoluble (lane 4) fractions were separated from these cells after 20 h by centrifugation after sonication. Each recombinant PDI family protein (arrowheads) was purified by His-tag column chromatography (lane 5) followed by gel filtration chromatography (lane 6). Proteins in each sample were separated by SDS-PAGE and stained with Coomassie Brilliant Blue. **(G)** The CD spectra of the purified recombinant TaPDIL1Aα (●), TaPDIL1B (○), TaPDIL2 (▲), TaPDIL3A (■), TaPDL4D (♦), and TaPDIL5A (Δ) were determined at a concentration of 0.3 mg/ml. **(H)** The purified recombinant TaPDIL1Aα (lane 1), TaPDIL1B (lane 2), TaPDIL2 (lane 3), TaPDIL3A (lane 4), TaPDIL4D (lane 5), or TaPDIL5A (lane 6) was detected by western blot analysis with anti-TaPDIL1Aα serum (lanes 1 and 2), anti-TaPDIL2 serum (lane 3), anti-TaPDIL3A serum (lane 4), anti-TaPDIL4D serum (lane 5), or anti-TaPDIL5A serum (lane 6). Asterisks indicate degradation products.

To investigate the enzymatic properties of wheat PDI family proteins, recombinant proteins without the putative N-terminal signal peptide were prepared from cDNAs of *TaPDIL1Aα*, *TaPDIL1B*, *TaPDIL2*, *TaPDIL3A*, *TaPDIL4D,* and *TaPDIL5A* using an *Escherichia coli* (*E. coli*) expression system, and then the proteins were purified (Figure 3A-F). All recombinant proteins were expressed as soluble proteins (Figure 3A-F) and eluted in a monomeric form from a gel filtration column (data not shown). All recombinant TaPDIL family proteins had circular dichroism (CD) spectra typical of well-folded α/β-type proteins (Figure 3G).

The oxidative refolding activities of these recombinant proteins were analyzed with reduced and denatured ribonuclease A (RNaseA) as a substrate. Both TaPDIL1Aα and TaPDIL1B exhibited higher activities than the other recombinant PDI family proteins (Table 3). Furthermore, the activities of TaPDIL1Aα and TaPDIL1B were approximately 1.5-fold higher than that of the recombinant soybean ortholog GmPDIL-1 [17] despite 63% share identity in amino acid sequence (Additional file 2: Figure S2). The activity of TaPDIL2 was nearly identical to that of soybean ortholog GmPDIL-2 [17], which has an amino acid sequence with 62% shared identity (Additional file 4: Figure S4). The recombinant TaPDIL3A had no oxidative refolding activity similar to soybean GmPDIL3 [19]. The lack of oxidative refolding activity of TaPDIL3A and GmPDIL-3 is thought to stem from their atypical active site motifs and the lack of a conserved arginine that is necessary for active site activity as described above. Furthermore, the activity of TaPDIL4D was approximately three- to four-fold higher than those of soybean orthologs, GmPDIS-1 and GmPDIS-2 [16], even though 75% and 74%, respectively, of the amino acid sequences are identical (Additional file 6: Figure S6). Finally, TaPDIL5A exhibited approximately four-fold greater activity than that of the soybean ortholog GmPDIM [18], which shares a 79% identical amino acid sequence (Additional file 7: Figure S7).

**Table 3:** **Oxidative refolding activity of recombinant wheat PDI family proteins**

**Wheat PDI family protein**	**Activity (mmol/min/mol)**	**Soybean PDI ortholog**	**Activity (mmol/min/mol)**
TaPDIL1Aα	736 ± 4	GmPDIL-1	460^b^
TaPDIL1B	665 ± 49		
TaPDIL2	287 ± 20	GmPDIL-2	259^b^
TaPDIL3A	N.D.	GmPDIL-3	N.D.^d^
TaPDIL4D	179 ± 7	GmPDIS-1	66^a^
		GmPDIS-2	43^a^
TaPDIL5A	175 ± 33	GmPDIM	45^c^

^a, b, c, d^Values are quoted from references 16, 17, 18, and 19, respectively.

Oxidative refolding activity was assayed using reduced and denatured RNaseA as a substrate.

### Expression of PDI family proteins in wheat caryopses

**Figure 4:**
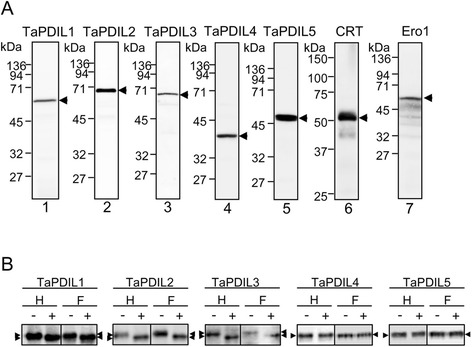
**Expression of wheat PDI family proteins in wheat caryopses. (A)** Proteins (25 μg) extracted from the caryopses at 25 (lanes 1, 3) or 15 (lanes 2, 4-7) dap were analyzed by western blot with serum against TaPDIL1Aα (lane 1), TaPDIL2 (lane 2), TaPDIL3A (lane 3), TaPDIL4D (lane 4), TaPDIL5A (lane 5), calreticulin (lane 6), or Ero1 (lane 7). **(B)** TaPDIL1, TaPDIL2, and TaPDIL3 are high mannose-type N-glycosylated proteins in wheat caryopses. The proteins extracted from the caryopses were treated with (+) or without (−) endoglycosidase H (H) or endoglycosidase F (F). The proteins (20 μg for TaPDIL1 and TaPDIL2; 30 μg for TaPDIL3, TaPDIL4, and TaPDIL5) were separated by SDS-PAGE and immunostained with serum against TaPDIL1Aα, TaPDIL2, TaPDIL3A, TaPDIL4D, or TaPDIL5A.

We generated antiserum using recombinant TaPDIL1Aα, TaPDIL2, TaPDIL3A, TaPDIL4D, or TaPDIL5A as an antigen. Each antiserum cross-reacted with the recombinant protein used for the immunization (Figure 3H). The antiserum against TaPDIL1Aα also cross-reacted with recombinant TaPDIL1B. The expression of each PDI family protein in wheat caryopses was confirmed by western blot analyses with these sera. The sera against TaPDIL1Aα, TaPDIL2, TaPDIL3A, TaPDIL4D, and TaPDIL5A recognized a 60-, 70-, 69-, 40-, and 47-kDa band, respectively, on gels containing proteins extracted from caryopses of bread wheat (Figure 4A). Because anti-TaPDIL1Aα serum cross-reacted with recombinant TaPDIL1B, a highly conserved protein categorized as group I PDI family proteins, it is likely that splicing variants of TaPDIL1Aα, TaPDIL1B, and TaPDIL1-3 [34] may all be detected by western blot analysis with this serum. Likewise, the sera against TaPDIL2, TaPDIL3A, TaPDIL4D, and TaPDIL5A were presumed to cross-react with their homolog categorized into group II, III, IV, or V, respectively. Thus, the proteins that cross-reacted with sera against TaPDIL1Aα, TaPDIL2, TaPDIL3A, TaPDIL4D, or TaPDIL5A were referred to as TaPDIL1, TaPDIL2, TaPDIL3, TaPDIL4, or TaPDIL5, respectively.

The sizes of the bands that cross-reacted with anti-TaPDIL4D and anti-TaPDIL5A sera approached the molecular sizes calculated from the amino acid sequences of TaPDL4D and TaPDIL5A, respectively, in the absence of the signal peptide. The sizes of bands that cross-reacted with the sera against TaPDIL1Aα, TaPDIL2, and TaPDIL3A were larger than the calculated molecular sizes based on the amino acid sequences of these PDI family proteins without the signal peptide, but these proteins contain one or two consensus N-glycosylation sites, suggesting that these PDI family proteins contain N-glycan(s). When caryopses proteins were digested with endoglycosidase H or F, the masses of the bands that were recognized by the sera against TaPDIL1Aα, TaPDIL2, and PDIL3A became smaller (Figure 4B), supporting the notion of N-glycosylation of these proteins in wheat caryopses. Because endoglycosidase H specifically cleaves high mannose-type oligosaccharides but not complex oligosaccharides from glycoproteins, these results indicate that the oligosaccharides attached to TaPDIL1, TaPDIL2, and TaPDIL3 are high mannose-type oligosaccharides. In agreement with these findings, TaPDIL1 purified from wheat was previously shown to be a glycoprotein [28]. In addition to PDI family proteins, we examined wheat orthologs of calreticulin and ER oxidoreductin 1 (Ero1) using western blot analyses. Calreticulin acts cooperatively with the human PDI family protein ER-60/ERp57 to fold monoglycosylated N-glycoproteins [47-50]. Ero1 is a PDI-activating enzyme located in the ER [51-54]. The active centers of PDI family proteins oxidize cysteine residues in substrate proteins and become a reduced form. PDI family proteins require other oxidizing molecules such as Ero1 for reoxidation of their active center cysteine residues. The sera against calreticulin and Ero1 cross-reacted with a 52-kDa band and 63-kDa band, respectively (Figure 4A). These band sizes were similar to those calculated from the putative amino acid sequences of the wheat orthologs of calreticulin (50,114; [EMS59406]) and Ero1 (65,619; [EMS58683]) [55].

**Figure 5:**
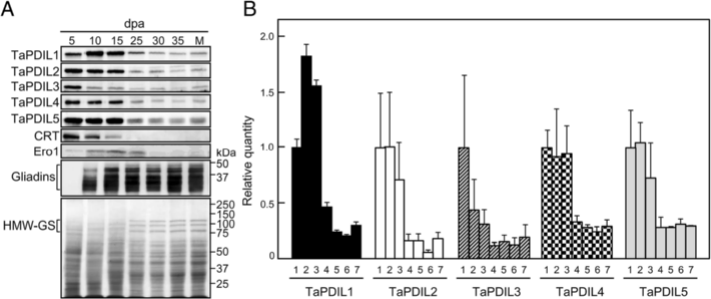
**Expression of wheat PDI family proteins in wheat caryopses during maturation. (A)** Proteins (25 μg) extracted from caryopses at 5 (lane 1), 10 (lane 2), 15 (lane 3), 25 (lane 4), 30 (lane 5), and 35 dpa (lane 6) as well as mature grain (M) were analyzed by western blot as described in Figure 4A. For detection of gliadins, anti-gliadin serum was used. Total proteins were stained with Coomassie Brilliant Blue (CBB). HMW-GS indicates high molecular glutenin subunit. **(B)** Levels of TaPDIL1, TaPDIL2, TaPDIL3, TaPDIL4, and TaPDIL5 in caryopses at 5 (1), 10 (2), 15 (3), 25 (4), 30 (5) and 35 dpa (6) and in mature grain (7) were estimated from the band intensities on western blots in A. Values were calculated as a ratio to the value obtained at 5 dpa. Data represent the mean ± standard error of three experiments.

The expression levels of the PDI family proteins, calreticulin, and Ero1 in the caryopses during maturation were determined by western blot analyses (Figure 5A). In addition, the levels of each of the PDI family proteins in bread wheat caryopses and flour were semi-quantitatively assessed by determining their band intensities on western blots (Figure 5B and Table 4). Levels of TaPDIL1 in the caryopses were highest at 10–15 dpa, which corresponds to the time of greatest synthesis of the seed storage proteins such as gliadins and glutenins (Figure 5A, Additional file 8: Figure S8). The levels of TaPDIL2, TaPDIL4, and TaPDIL5 in caryopses were also highest at 5–15 dpa in the maturing period. The level of TaPDIL3 in the caryopses was highest at 5 dpa and then decreased with time. The content of TaPDIL1 was the highest among PDI family proteins (Table 4). The expression of *TaPDIL1* mRNA in the developing caryopses has been reported to be the highest of those of PDI family proteins [34]. The expression of wheat Ero1 was highest at 15 dpa (Figure 5A). Wheat Ero1 may activate wheat PDI family proteins, but whether plant Ero1 activates plant PDI family proteins *in vivo* remains unproven. Knockdown of rice Ero1 (OsERO1) revealed that native disulfide bond formation in proglutelins depends on an electron transfer pathway involving OsERO1 [56]. Wheat calreticulin was also highly expressed during the period of grain filling (Figure 5A). Levels of all PDI family proteins, calreticulin, and Ero1 were drastically diminished in the late maturing period (25–35 dpa; Figure 5A and B) when grain weights decreased due to desiccation (Additional file 8: Figure S8); however, low levels of PDI family proteins remained even in the mature grains. Calreticulin and Ero1 were not detected in the 25–35 dpa caryopses or in the mature grains.

**Table 4:** **PDI family proteins in wheat caryopses and flour**

**Wheat PDI family protein**	**Content (pmol/mg protein)**							
	**5 dpa**	**10 dpa**	**15 dpa**	**25 dpa**	**30 dpa**	**35 dpa**	**Mature**	**flour**
TaPDIL1	15.0	27.4	23.2	6.9	3.4	2.9	4.3	7.3
TaPDIL2	3.9	3.8	2.7	0.5	0.6	0.4	0.6	1.1
TaPDIL3	0.7	0.3	0.2	0.1	0.1	0.1	0.1	0.1
TaPDIL4	12.7	11.6	12.1	4.1	3.2	3.0	3.5	1.5
TaPDIL5	1.5	1.3	1.2	0.3	0.2	0.2	0.3	0.3

Contents by weight of PDI family proteins were estimated from the band intensities on western blot analysis of the caryopses (Figure 4B) and flour (Haruyokoi) with recombinant TaPDIL1Aα, TaPDIL2, TaPDIL3A, TaPDIL4D, and TaPDIL5A used as standard proteins for determination of TaPDIL1, TaPDIL2, TaPDIL3, TaPDIL4, and TaPDIL5, respectively. Molar quantities of TaPDIL1, TaPDIL2, TaPDIL3, TaPDIL4, and TaPDIL5 included in 1 mg of caryopses protein or flour protein were calculated with each molecular weight of TaPDIL1Aα, TaPDIL2, TaPDIL3A, TaPDIL4D, and TaPDIL5A (as shown in Table 2), respectively.

### Localization of PDI family proteins in wheat caryopses

**Figure 6:**
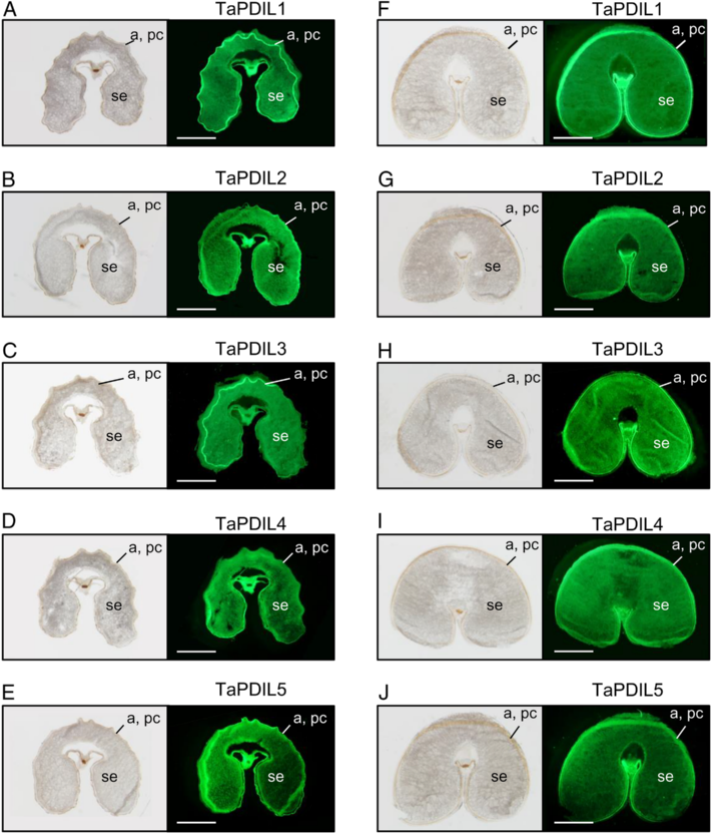
**Distribution of wheat PDI family proteins in immature wheat caryopses.** Cross sections of caryopses at 10 dpa **(A-E)** and 20 dpa **(F-J)** were immunostained with serum against TaPDIL1Aα **(A, F)**, TaPDIL2 **(B, G)**, TaPDIL3A **(C, H)**, TaPDIL4D **(D, I)**, or TaPDIL5A **(E, J)**. Specimens were observed with a stereomicroscope SZX16. Fluorescent images are shown on the right panels in **(A-J)**. Visible light images collected simultaneously are shown on the left in **A**-**J**. se, starchy endosperm; a, aleurone layer; pc, pericarp. Scale bar = 1 mm.

Localization of each PDI family protein in the immature wheat caryopses at 10 and 20 dpa was investigated by immunofluorescence microscopy. The antisera for PDI family proteins were confirmed to specifically react with their respective native recombinant PDI family protein (Additional file 9: Figure S9). The aleurone cells, pericarp, and starchy endosperm of the caryopsis were labeled with the sera raised against TaPDIL1Aα, TaPDIL2, TaPDIL3A, TaPDIL4D, or TaPDIL5A (Figure 6). These distributions suggest that these PDI family proteins are required for folding nascent proteins that are synthesized *de novo* in these tissues during grain development.

**Figure 7:**
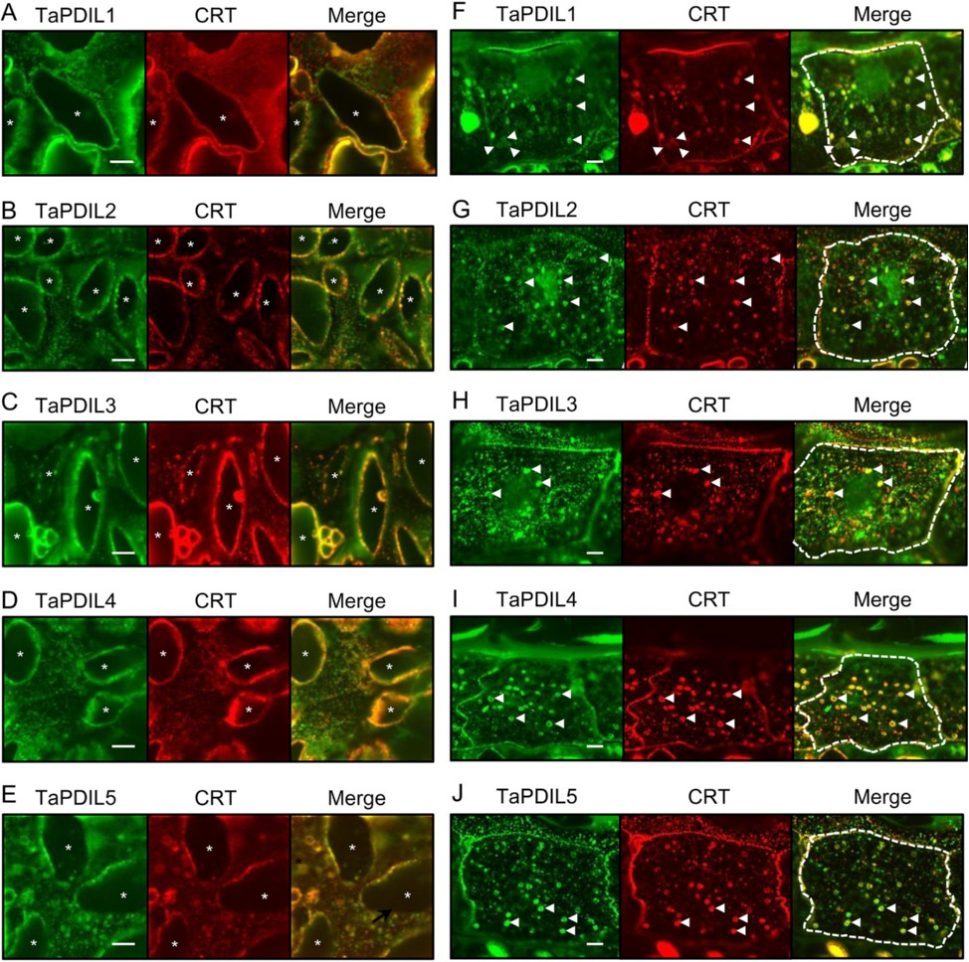
**Subcellular distribution of wheat PDI family proteins in immature starchy endosperm and aleurone cells.** Cross sections of caryopses at 10 dpa were immunostained with a combination of sera against TaPDIL1Aα (**A** and **F**, green), TaPDIL2 (**B** and **G**, green), TaPDIL3A (**C** and **H**, green), TaPDIL4D (**D** and **I**, green), or TaPDIL5A (**E** and **J**, green) and calreticulin (CRT, red). Specimens were observed with a Confocal Imaging System FV1200. Merged images of red and green are also shown. Endosperm sections and aleurone cells are shown in the left and right panels, respectively. Asterisks indicate A-type starch granules. Arrowheads indicate aleurone grains. White dotted lines show the profile of one aleurone cell. Scale bar = 5 μm.

The TaPDIL1 group proteins, TaPDIL2, TaPDIL3A, and TaPDIL5A contain putative signal peptides as well as the C-terminal ER retention signal. Therefore, these PDI family proteins were expected to localize to the ER. In addition, the ER-localization of TaPDIL4D, which has an N-terminal signal peptide but no C-terminal ER retention signal, was also predicted because soybean orthologs GmPDIS-1 and GmPDIS-2 localize to the ER [16]. The ER-localization of all PDI family proteins in the starchy endosperm was indicated by their network-like distribution in the cytoplasm and co-localization with calreticulin, a well-known ER luminal protein (Figure 7A-E). In the starchy endosperm, peripheral regions of A-type starch granules were strongly immunostained with anti-PDI family and anti-calreticulin sera, suggesting that the ER was densely packed around the starch granules. The existence of all wheat PDI family proteins in the ER of the starchy endosperm cells during grain filling suggests that these factors play important roles in the folding of storage proteins such as gliadins and glutenins, which are synthesized and folded in the ER. γ-Gliadin has been shown to require PDI family proteins for folding by *in vitro* translational experiment [5]. In the aleurone cells, members of all five groups of PDI family proteins were labeled along with calreticulin in the periphery of aleurone grains and network structure (Figure 7F-J), suggesting localization of these proteins to the ER. In aleurone grains, storage proteins accumulate during seed development [57]. These PDI family proteins may assist in the folding of storage proteins synthesized in the ER of the aleurone cells, and the resultant folded storage proteins accumulate in the aleurone grains. However, the specific details of the transport of storage proteins from the ER to aleurone grains remains unclear. In addition, the five groups of PDI family proteins were also detected at the boundaries of aleurone cells. The localization mechanism and physiological roles of those PDI family proteins at the aleurone cell boundary remain to be elucidated.

**Figure 8:**
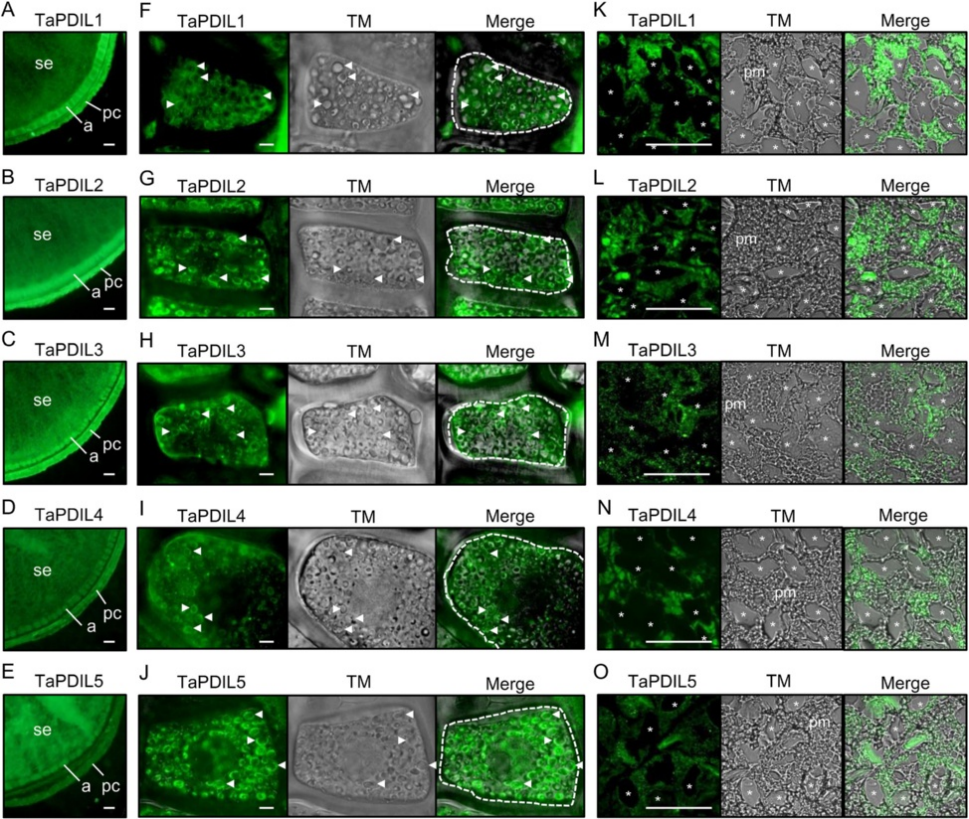
**Distribution of wheat PDI family proteins in the mature aleurone cells and the protein matrix of wheat grains.** Cross sections of mature grains were immunostained with sera against TaPDIL1Aα **(A, F, K)**, TaPDIL2 **(B, G, L)**, TaPDIL3A **(C, H, M)**, TaPDIL4D **(D, I, N)**, or TaPDIL5A **(E, J, O)**. Specimens were observed with a fluorescence microscope BZ-9000 **(A-E, K-O)** and a Confocal Imaging System FV1200 **(F-J)**. Visible light images (gray) collected simultaneously are shown in the middle panels **(F-O)**. Merged fluorescent and visible light images are also shown in the right panels **(F-O)**. a, aleurone layer; pc, pericarp; pm, protein matrix; se, starchy endosperm. Arrowheads indicate aleurone grains. White dotted lines show the profile of one aleurone cell. Asterisks indicate A-type starch granules. Scale bars in **A**-**E** = 250 μm. Scale bars in **F**-**J** = 5 μm. Scale bars in **K**-**O** = 50 μm.

In the mature grains, the members of the five groups of PDI family proteins were labeled in the aleurone cells and starchy endosperm (Figure 8A-E). In the aleurone cells of the mature wheat grain, the ER has been found in two regions: the periphery of the aleurone grain-spherosome complex and the cytoplasm as spread network structures [58]. The periphery of the aleurone grain-spherosome complex was mainly labeled with antisera to the five anti-PDI family proteins and calreticulin (Figure 8F-J). Based on these findings, we predict that the PDI family proteins that localized in the aleurone cells of the mature grain accumulated prior to grain formation and function in folding newly synthesized hydrolytic enzymes in the ER during germination. In mature cereal grain endosperm, only aleurone cells are alive [59]. When cereal grains imbibe water, α-amylases and cysteine proteinases, which may contain intramolecular disulfide bonds [60,61], are synthesized in the ER of the aleurone cells in response to gibberellic acid secretion into the endosperm from the embryo via the scutellum [62,63]. These hydrolytic enzymes are secreted to the starchy endosperm to break down starch and protein that accumulated in the starchy endosperm to supply carbon and nitrogen used in germination. Whereas the secretion of α-amylases and cysteine proteases from the aleurone layers increased in response to gibberellic acid treatment for 24 h, the level of the barley TaPDIL-1 ortholog was not affected by gibberellic acid treatment [64], suggesting that the barley TaPDIL-1 ortholog that remains in the aleurone cell of the wheat grain may help fold the newly synthesized hydrolytic enzymes.

During the late stage of maturation, the starchy endosperm cells undergo programmed cell death, and the protein bodies fuse to the protein matrix [65]. The analyzed members of the five groups of PDI family proteins were distributed throughout the fused protein matrix (Figure 8K-O). Because wheat flour arises from milled starchy endosperm, the five groups of PDI family proteins must be present in flour (Table 4). We previously detected oxidative refolding activity in an extract from wheat flour, and its activity was inhibited by the PDI inhibitor bacitracin [66]. Therefore, PDI family proteins may be active in both the starchy endosperm of the dry mature grain and flour. It is unclear whether the PDI family proteins that remain in the starchy endosperm of the wheat grain play a physiological role during germination. Further studies are needed to identify the substrate proteins associated with each PDI family protein *in vivo* and to determine the effects of knockdown of these factors on wheat phenotypes, such as seed storage protein accumulation and plant body growth. Furthermore, PDI family proteins may affect the quality of food products. We previously demonstrated that PDI family proteins play a role in retaining glutenin macropolymers during dough mixing, and these polymers are the most important factor for dough’s tensile strength [66]. We semi-quantitatively analyzed the levels of the five PDI family protein groups in a wheat flour sample (Table 4) and found that among the five groups of PDI family proteins, TaPDIL1 levels were the highest. Because the oxidative refolding activities of recombinant TaPDIL1A and TaPDIL1B were the strongest of the five recombinant PDI family proteins, TaPDIL1 may primarily function in retaining glutenin macropolymers during dough mixing. Therefore, PDI family proteins may be an important contributing factor to the quality wheat flour, and they may serve as targets for genetic manipulation in the future.

## Conclusions

In eukaryotes, PDI and PDI family proteins function in multiple essential capacities such as oxidative folding of nascent proteins, molecular chaperoning, antigen presentation, degradation of abnormal proteins, and redox signaling. Most information about such functions has been obtained from mammalian cell studies. The physiological functions and functional mechanisms of plant PDI family proteins remain largely unknown. Here, we demonstrated that four of the five groups of wheat PDI family proteins exhibit oxidative refolding activity on reduced and denatured RNaseA. All five groups of PDI family proteins were highly expressed throughout the caryopses and in particular, in the starchy endosperm during grain filling. As expected from their primary structures, these proteins localized to the ER. These results suggest that PDI family proteins play an important role in folding seed storage proteins synthesized in the ER. In mature grain, the PDI family proteins from all five groups remain in the ER surrounding the aleurone grains of the aleurone cells and the protein matrix of the starchy endosperm. Because many of the hydrolytic enzymes are synthesized and folded in the ER of the aleurone cells and secreted to the starchy endosperm to break down starch and storage proteins during the early stage of germination, these PDI family proteins may function in folding nascent hydrolytic enzymes. In conclusion, our work provides significant basic information about the physiological functions of these PDI family proteins during the wheat life cycle. In the future, it will be necessary to identify the *in vivo* substrate proteins and determine the effects of selective silencing of PDI family genes on wheat phenotypes with respect to seed storage protein folding, transport, and accumulation; seed germination; and plant body growth.

## Methods

### Plant material

Bread wheat plants were grown in an open field at Kyoto University. The developing caryopses were collected 147 days after seeding bread wheat plants (*Triticum aestivum* cv. Yumeshiho) for isolation of mRNA to clone wheat PDI family cDNAs. The developing caryopses from 5–35 dpa were collected from *Triticum aestivum* cv. Haruyokoi at 5-day intervals for western blot analysis and immunohistochemistry. The caryopses were frozen in liquid nitrogen and stored at −80°C immediately after harvest.

### cDNA cloning

The cloning of TaPDIL1, TaPDIL2, TaPDIL3, TaPDIL4, and TaPDIL5 cDNAs was performed using RT-PCR. Total RNA was isolated using the Sepasol RNA I (Nacalai Tesque, Kyoto, Japan) reagent according to the manufacturer’s protocol. The amplification of cDNA from total RNA was performed with a Prime Script II RTase (TaKaRa Bio Inc., Shiga, Japan) using the following oligonucleotide primers: 5’-CATGGCGATCTCCAAGGTC-3’ and 5’-CTCTACCTGTCTGCTGCTAGC-3’ for TaPDIL1 (GenBank:AJ277379), 5’-TGGACTGAATTCGGCGGATCCATTTCCACTCC CACTTCCCCCAACG-3’ and 5’-CATCCCCTGGTTTCGGCGTCGGCTC-3’ for TaPDIL2 (GenBank:FN555316), 5’-TGGACTGAATTCCCCTCTCCTAGATCTCGGAGGAGGAGCGC-3’ and 5’-TGGACTGAATTCATGGCTACTGCGTAACCGTGACCAACCCCTAC-3’ for TaPDIL3 (GenBank:FN555317), 5’-TGGACTCTCGAGGTGCAAGAAGAACAGGTGCCAACCG-3’ and 5’-TGGACTCTCGAGCCGCTAAACTTTCACTGCCATCTCTCTGATCTC-3’ for TaPDIL4 (GenBank:FN555318), and 5’-TGGACTAAGCTTCCGGCTTCCAGAAATTTTTCAACGACGC-3’ and 5’-TGGACTAAGCTTCCACCTTGCACATCAGAGCTTTCTCCCAC-3’ for TaPDIL5 (GenBank:FN555320). Nested PCRs were performed with the DNA fragments amplified by the RT-PCR as a template using the following oligonucleotide primers: 5’-ATGGCGATCTCCAAGGTCTGGATC-3’ and 5’- CTGCTGCTAGCAAGACTGATGC-3’ for TaPDIL1, 5’-CCATGGCGGCGATGCCGATGC-3’ and 5’-GGCTCCTACTTGTTGTCAATGGTG-3’ for TaPDIL2, 5’-GGAGGAGCGCGATGAGGGCGACG-3’ and 5’-CATTTTCCTTCAACGCGGCCAGC-3’ for TaPDIL3, 5’-GGTCA CCCGAGCTCGCAG-3’ and 5’-CTTCACT TCTCTCTTGTGGC-3’ for TaPDIL4, and 5’-TGGACTAAGCTTCCGGCTTCCAGAAATTTTTCAACGACGC-3’ and 5’-CACATCAGAGCAAGTGAAGC-3’ for TaPDIL5. The amplified TaPDIL1, TaPDIL2, and TaPDIL3 DNA fragments were subcloned into pCR Blant II-TOPO (Invitrogen, Carlsbad, CA) and transformed in *E. coli* DH5. The amplified TaPDIL4 and TaPDIL5 DNA fragments were subcloned into T-Vector pMD20 (TaKaRa Bio Inc.) and transformed into *E. coli* DH5 cells. The inserts in the plasmid vectors were sequenced using the fluorescence dideoxy chain termination method with an ABI PRISM® 3100-Avant Genetic Analyzer (Applied Biosystems, Foster City, CA).

### Construction of His-tagged expression plasmids

Expression plasmids encoding mature His-tagged TaPDIL1Aα, TaPDIL1B, TaPDIL2, TaPDIL3A, TaPDIL4D, and TaPDIL5A without the putative signal peptide were constructed. Briefly, the DNA fragment was amplified from the cDNA of TaPDIL1Aα, TaPDIL1B, TaPDIL2, TaPDIL3A, TaPDIL4D, and TaPDIL5A by PCR using the following oligonucleotide primers: 5’-GACGACGACAAGATG GAGGAGGCCGCCGCCGCCGAGGAG-3’ and 5’-GAGGAGAAGCCCGGTCAGAGCTCGTCCTTCAGAGGCTC-3’ for TaPDIL1Aα and TaPDIL1B, 5’-GACGACGACAAGATGGCAGTCCCCACCTCCAACCCGG-3’ and 5’-GAGGAGAAGCCCGGTCACAACTCGTCCTTCGGGTTCGAAC-3’ for TaPDIL2, 5’-GACGACGACAAGATG GCGAAGCTCGATCTGGACGAGGTG-3’ and 5’-GAGGAGAAGCCCGGTCATAGCTCATCCTTGACATTGTC-3’ for TaPDIL3A, 5’-GACGACGACAAGATGGACGAGGTGCTTGCCCTCACGGAG-3’ and 5’-GAGGAGAAGCCCGGTCAAGAGGAGAAGGCTGAAAGAATG-3’ for TaPDIL4D, and 5’-GACGACGACAAGATGCTCTACTCCGCCGGCTCCCCGGTC-3’ and 5’-GAGGAGAAGCCCGGTCACAACTCGTCGTTGGGCGCAGAG-3’ for TaPDIL5A. The amplified DNA fragment was subcloned into the ligation-independent cloning site of the pET46Ek/LIC vector (EMD Biosciences, Inc., San Diego, CA). The recombinant proteins have a His-tag linked to the amino terminus.

### Expression and purification of recombinant wheat PDI family proteins

*E. coli* BL21(DE3) cells were transformed with the expression vectors as described above. The expression of recombinant proteins was induced by the addition of 0.4 mM isopropyl thiogalactoside at 30°C for 20 h. All expressed recombinant proteins were soluble in *E. coli*. The cells from 1 L culture broth were collected by centrifugation, washed twice by suspending in phosphate-buffered saline and centrifugation, disrupted by sonication in 8 mL of 20 mM Tris (hydroxymethyl) aminomethane (Tris)-HCl buffer (pH 8) containing 5 mM imidazole and 0.5 M NaCl (binding buffer), and then centrifuged at 10,000 × g for 30 min at 4°C. The supernatant was filtered through a Millex Syringe-driven filter 33 mm (Millipore Corporation, Billerica, MA) and applied to a column packed with Ni Sepharose 6 Fast Flow (GE Healthcare, Piscataway, NJ). After washing the column with binding buffer containing 60 mM imidazole, recombinant proteins were eluted with binding buffer containing 1 M imidazole, concentrated with a Vivaspin 20 (GE Healthcare), and then subjected to gel filtration chromatography on a TSK gel G3000SW column (Tosoh, Tokyo, Japan) equilibrated with 20 mM Tris–HCl buffer (pH 7.4) containing 0.15 M NaCl and 10% glycerol. Recombinant proteins that eluted in the inside volume fractions were collected. The concentrations of purified recombinant proteins were determined from their absorbance at 280 nm using the molar extinction coefficients calculated with the modified method of Gill and von Hippel [67]. Extinction coefficients of 43,820, 42,330, 40,840, 33,265, 36,620, and 54,275 M^−1^ cm^−1^ were used for recombinant TaPDIL1Aα, TaPDIL1B, TaPDIL2, TaPDIL3A, TaPDIL4D, and TaPDIL5A, respectively.

The CD spectra of recombinant proteins in 20 mM Tris–HCl buffer (pH 7.4) containing 150 mM NaCl and 10% glycerol were obtained using a J-720 spectropolarimeter (JASCO Corp., Tokyo, Japan) in a 1-mm path-length cell with a scan speed of 20 nm/min at 14°C.

### RNaseA refolding assay

Oxidative refolding activity was assayed by measuring the RNase activity produced through the regeneration of the active form from reduced and denatured RNaseA that was prepared as described previously by Creighton [68]. Each reaction mixture comprised 180 mM [4-(2-hydroxyethyl)-1-piperazinyl]ethanesulfonic acid (pH 7.5), 150 mM NaCl, 2 mM CaCl_2_, 0.5 mM glutathione disulfide, 2 mM glutathione, 1 mg/mL reduced RNaseA, and 0.25 μM recombinant wheat PDI family proteins. The reaction mixture was incubated at 25°C. An aliquot (16 μl) of the reaction mixture was removed, and RNaseA activity was measured spectrophotometrically at 284 nm with cytidine2’:3’-cyclic monophosphate as the substrate [69]. Reactivation of reduced RNaseA in the absence of recombinant protein was subtracted from reactivation in the presence of recombinant wheat PDI family proteins.

### Antibodies

Antisera were prepared by Japan Bio Serum (Hiroshima, Japan). Purified recombinant TaPDIL1Aα, TaPDIL2, TaPDIL3A, TaPDIL4D, and TaPDIL5A were emulsified with Freund’s complete adjuvant and intradermally injected into female guinea pigs. Anti-calreticulin and anti-Ero1 guinea pig sera were prepared with recombinant soybean calreticulin and soybean Ero1 expressed in *E. coli* as described previously [16]. Anti-gliadin rabbit serum was purchased from Sigma-Aldrich. Inc. (St. Louis, MO).

### Western blot analysis

Wheat caryopses that had been frozen in liquid nitrogen were ground into fine powders with a Multi-beads Shocker (Yasui Kikai. Osaka, Japan) [70]. Proteins were extracted from ground caryopses by boiling for 5 min in sodium dodecyl sulfate -polyacrylamide gel electrophoresis (SDS-PAGE) sample buffer [71] containing a 1% cocktail of protease inhibitors (Sigma-Aldrich). Debris in the sample was removed by centrifugation at 5000 × *g* for 20 min. The concentrations of proteins in the samples were measured with a protein assay kit (RC DC protein assay, Bio-Rad Laboratories, Hercules, CA). To cleave the N-glycan modifications of the proteins by endoglycosidase H (Sigma-Aldrich) or endoglycosidase F (Sigma-Aldrich), proteins were extracted from the caryopses in 2% SDS, 0.1 M Tris–HCl (pH 8.6), 1% Nonidet P-40. The extract was diluted 20-fold for glycosidase H in 0.1 M Tris–HCl (pH 8.6)/1% Nonidet P-40 or for glycosidase F in 50 mM potassium phosphate buffer (pH 5.5) containing 0.25% 2-mercaptoethanol. Proteins (0.4 mg) were treated with 10 mU endoglycosidase H or 125 mU endoglycosidase F at 37°C for 16 h. Proteins were subjected to SDS-PAGE [71] and blotted onto polyvinylidene difluoride membranes. The proteins were then immunostained with the specific guinea pig or rabbit antisera as primary antibodies and horseradish peroxidase-conjugated anti-guinea pig IgG serum (Promega Corporation, Madison, WI) or anti-rabbit IgG serum (Santa Cruz Biotechnology, Inc., Dallas, TX) as secondary antibodies. The bands were visualized using the Western Lightning Plus-ECL Enhanced Chemiluminescence Substrate (Perkin Elmer Life Sciences, Boston, MA).

### Semi-quantitative assay of wheat PDI family proteins in caryopses

Proteins extracted from the developing caryopses were separated along with known amounts of recombinant PDI family proteins on the same gel by SDS-PAGE and detected by western blot analysis as described above. The amount of each PDI family protein was calculated from the band intensities of the sample and the corresponding recombinant protein. Recombinant TaPDIL1Aα, TaPDIL2, TaPDIL3A, TaPDIL4D, and TaPDIL5A were used as standards for the semi-quantitative analysis of TaPDIL1, TaPDIL2, TaPDIL3, TaPDIL4, and TaPDIL5, respectively.

### Microscopic observation

Caryopses from developing and mature grains were cut and fixed with 4% paraformaldehyde in 80 mM piperazine-1,4-bis (2-ethanesulfonic acid) at pH 6.5, 5 mM ethylene glycol tetraacetic acid, and 2 mM MgCl_2_ for 2 h at room temperature. The fixed caryopses were dehydrated with a series of 50, 50, 70, 70, 80, 90, 95, and 99.5% ethanol for 40 min each at room temperature. The dehydrated caryopses were embedded in Historesin (Leica Microsystems, Wetzlar, Germany) and sliced into sections with a rotary microtome PR-50 (Yamato Kohki Industrial Co., Ltd., Saitama, Japan). The sections were mounted on glass slides and stained with guinea pig antisera against TaPDIL1Aα, TaPDIL2, TaPDIL3A, TaPDIL4D, and TaPDIL5A and subsequently with a secondary antibody, which was Cy3-conjugated anti-guinea pig IgG goat serum (Chemicon International, Temecula, CA). For detection of calreticulin, specimens were stained with an anti-calreticulin rabbit serum followed by a biotin-anti-rabbit IgG goat serum (Cortex Biochem, San Leandro, CA) and incubation with Cy5-streptavidin (GE Healthcare Bio-Sciences Corp.). The specimens were examined on a stereomicroscope SZX16 equipped with an SZX2-FGFP filter and a DP73 camera (Olympus Co., Tokyo, Japan), a Confocal Imaging System FV1200 (Olympus), and a fluorescence microscope BZ-9000 (Keyence Corp., Osaka, Japan).

## Additional files

Additional file 1: Figure S1. Alignment of amino acid sequences of TaPDIL1Aα, TaPDIL1Aβ, TaPDIL1Aγ, TaPDIL1Aδ, and TaPDIL1B. Additional file 2: Figure S2. Alignment of amino acid sequences of TaPDIL1Aα and soybean GmPDIL-1. Additional file 3: Figure S3. Alignment of amino acid sequences of TaPDIL2 and TaPDIL2-1. Additional file 4: Figure S4. Alignment of amino acid sequences of TaPDIL2 and soybean GmPDIL-2. Additional file 5: Figure S5. Alignment of amino acid sequences of TaPDIL3A and soybean GmPDIL-3a. Additional file 6: Figure S6. Alignment of amino acid sequences of TaPDIL4D, soybean GmPDIS-1, and soybean GmPDIS-2. Additional file 7: Figure S7. Alignment of amino acid sequences of TaPDIL5A and soybean GmPDIM. Additional file 8: Figure S8. Caryopsis weight after anthesis. Additional file 9: Figure S9. Dot blot analysis of recombinant wheat PDI family proteins.

## Abbreviations

CD: Circular dichroism
dpa: Days post anthesis
ER: Endoplasmic reticulum
Ero1: ER oxidoreductin 1
*Escherichia coli*: *E. coli*
PDI: protein disulfide isomerase
RNaseA: Ribonuclease A
SDS-PAGE: Sodium dodecyl sulfate-polyacrylamide gel electrophoresis
Tris: Tris (hydroxymethyl) aminomethane
