# Supplementary material for: Expression and characterization of protein disulfide isomerase family proteins in bread wheat
Source: BMC Plant Biol. 2015 Mar 4;15:73. doi: 10.1186/s12870-015-0460-2 (PMC4355359; doi:10.1186/s12870-015-0460-2)
Supplement: Additional file 1: Figure S1. — Alignment of amino acid sequences of TaPDIL1Aα, TaPDIL1Aβ, TaPDIL1Aγ, TaPDIL1Aδ, and TaPDIL1B. [file 12870_2015_460_MOESM1_ESM.pdf]

```
TaPDIL1Aα     1 MAISKVWISL LLALAVVLSA PAARAEEAAA AEEAAAAPEA VLTLHADNFD DAIAKHPFIL  60
TaPDIL1Aβ     1 MAISKVWISL LLALAVVLSA PAAS------ --------EA VLTLHADNFD DAIAKHPFIL  46
TaPDIL1Aγ     1 MAISKVWISL LLAL------ ---------- ---------- --TLHADNFD DAIAKHPFIL  32
TaPDIL1Aδ     1 MAISKVWISL LL-------- ---------- ---------- --TLHADNFD DAIAKHPFIL  30
TaPDIL1B      1 MAISKVWISL LLALAVVLSA PAARAEEAAA AEEAAAAPEA VLTLHADNFD DAIAKHPFIL  60
                ********** **                                ******** **********

TaPDIL1Aα    61 VEFYAPWCGH CKSLAPEYEK AAQLLSKHDP AIVLAKVDAN DEKNKPLAGK YEVQGFPTLK 120
TaPDIL1Aβ    47 VEFYAPWCGH CKSLAPEYEK AAQLLSKHDP AIVLAKVDAN DEKNKPLAGK YEVQGFPTLK 106
TaPDIL1Aγ    33 VEFYAPWCGH CKSLAPEYEK AAQLLSKHDP AIVLAKVDAN DEKNKPLAGK YEVQGFPTLK  92
TaPDIL1Aδ    31 VEFYAPWCGH CKSLAPEYEK AAQLLSKHDP AIVLAKVDAN DEKNKPLAGK YEVQGFPTLK  90
TaPDIL1B     61 VEFYAPWCGH CKSLAPEYEK AAQLLSKHDP AIVLAKVDAN DEKNKPLAGK YEVQGFPTLK 120
                ********** ********** ********** ********** ********** **********

TaPDIL1Aα   121 IFRNGGKNIQ EYKGPREAEG IVEYLKKQVG PASKEIKAPE DATYLEDGKI HIVGVFTEFS 180
TaPDIL1Aβ   107 IFRNGGKNIQ EYKGPREAEG IVEYLKKQVG PASKEIKAPE DATYLEDGKI HIVGVFTEFS 166
TaPDIL1Aγ    93 IFRNGGKNIQ EYKGPREAEG IVEYLKKQVG PASKEIKAPE DATYLEDGKI HIVGVFTEFS 152
TaPDIL1Aδ    91 IFRNGGKNIQ EYKGPREAEG IVEYLKKQVG PASKEIKAPE DATYLEDGKI HIVGVFTEFS 150
TaPDIL1B    121 IFRNGGKNIQ EYKGPREAEG IVEYLKKQVG PASKEIKAPE DATYLEDGKI HIVGVFTEFS 180
                ********** ********** ********** ********** ********** **********

TaPDIL1Aα   181 GTEFTNFLEL AEKLRSDYDF GHTVHANHLP RGDAAVERPL VRLFKPFDEL VVDSKDFDVS 240
TaPDIL1Aβ   167 GTEFTNFLEL AEKLRSDYDF GHTVHANHLP RGDAAVERPL VRLFKPFDEL VVDSKDFDVS 226
TaPDIL1Aγ   153 GTEFTNFLEL AEKLRSDYDF GHTVHANHLP RGDAAVERPL VRLFKPFDEL VVDSKDFDVS 212
TaPDIL1Aδ   151 GTEFTNFLEL AEKLRSDYDF GHTVHANHLP RGDAAVERPL VRLFKPFDEL VVDSKDFDVS 210
TaPDIL1B    181 GTEFTNFLEV AEKLRSDYDF GHTVHANHLP RGDAAVERPL VRLFKPFDEL VVDSKDFDVS 240
                *********  ********** ********** ********** ********** **********

TaPDIL1Aα   241 ALEKFIDASS TPKVVTFDKN PDNHPYLLKY FQSNAPKAML FLNFSTGPFE SFKSAYYGAV 300
TaPDIL1Aβ   227 ALEKFIDASS TPKVVTFDKN PDNHPYLLKY FQSNAPKAML FLNFSTGPFE SFKSAYYGAV 286
TaPDIL1Aγ   213 ALEKFIDASS TPKVVTFDKN PDNHPYLLKY FQSNAPKAML FLNFSTGPFE SFKSAYYGAV 272
TaPDIL1Aδ   211 ALEKFIDASS TPKVVTFDKN PDNHPYLLKY FQSNAPKAML FLNFSTGPFE SFKSAYYGAV 270
TaPDIL1B    241 ALEKFIEASS TPKVVTFDKN PDNHPYLLKF FQSNAPKAML FLNFSTGPFE SFKKAYYGAV 300
                ****** *** ********** *********  ********** ********** *** ******

TaPDIL1Aα   301 EEFSGKDVKF LIGDIEASQG AFQYFGLKED QAPLILIQDS DSKKFLKEQV EAGQIVAWLK 360
TaPDIL1Aβ   287 EEFSGKDVKF LIGDIEASQG AFQYFGLKED QAPLILIQDS DSKKFLKEQV EAGQIVAWLK 346
TaPDIL1Aγ   273 EEFSGKDVKF LIGDIEASQG AFQYFGLKED QAPLILIQDS DSKKFLKEQV EAGQIVAWLK 332
TaPDIL1Aδ   271 EEFSGKDVKF LIGDIEASQG AFQYFGLKED QAPLILIQDS DSKKFLKEQV EAGQIVAWLK 330
TaPDIL1B    301 EEFSGKDVKF LIGDIEASQG AFQYFGLKED QAPLILIQDS DSKKFLKEQV EAGQIVAWLK 360
                ********** ********** ********** ********** ********** **********

TaPDIL1Aα   361 DYFDGKLTPF RKSEPIPEAN NEPVKVVVAD NIHDVVFKSG KNVLIEFYAP WCGHCKKLAP 420
TaPDIL1Aβ   347 DYFDGKLTPF RKSEPIPEAN NEPVKVVVAD NIHDVVFKSG KNVLIEFYAP WCGHCKKLAP 406
TaPDIL1Aγ   333 DYFDGKLTPF RKSEPIPEAN NEPVKVVVAD NIHDVVFKSG KNVLIEFYAP WCGHCKKLAP 392
TaPDIL1Aδ   331 DYFDGKLTPF RKSEPIPEAN NEPVKVVVAD NIHDVVFKSG KNVLIEFYAP WCGHCKKLAP 390
TaPDIL1B    361 DYFDGKLTPF RKSEPIPEAN NEPVKVVVAD NVHDVVFKSG KNVLIEFYAP WCGHCKKLAP 420
                ********** ********** ********** * ******** ********** **********

TaPDIL1Aα   421 ILDEAAATLQ SEEDVVIAKI DATANDVPGE FDVQGYPTLY FVTPSGKKVS YEGGRTADEI 480
TaPDIL1Aβ   407 ILDEAAATLQ SEEDVVIAKI DATANDVPGE FDVQGYPTLY FVTPSGKKVS YEGGRTADEI 466
TaPDIL1Aγ   393 ILDEAAATLQ SEEDVVIAKI DATANDVPGE FDVQGYPTLY FVTPSGKKVS YEGGRTADEI 452
TaPDIL1Aδ   391 ILDEAAATLQ SEEDVVIAKI DATANDVPGE FDVQGYPTLY FVTPSGKKVS YEGGRTADEI 450
TaPDIL1B    421 ILDEAAATLQ SEEDVVIAKM DATANDVPSE FDVQGYPTLY FVTPSGKKVS YEGGRTADEI 480
                ********** *********  ******** * ********** ********** **********

TaPDIL1Aα   481 VDYIKKNKET AGQAAAAATE KAAEPAATEP LKDEL 515
TaPDIL1Aβ   467 VDYIKKNKET AGQAAAAATE KAAEPAATEP LKDEL 501
TaPDIL1Aγ   453 VDYIKKNKET AGQAAAAATE KAAEPAATEP LKDEL 487
TaPDIL1Aδ   451 VDYIKKNKET AGQAAAAATE KAAEPAATEP LKDEL 485
TaPDIL1B    481 VDYIKKNKET AGQ---AATE KAAEPAATEP LKDEL 512
                ********** ***   **** ********** *****
```

Figure S1. Alignment of amino acid sequences of TaPDIL1Aα, TaPDIL1Aβ, TaPDIL1Aγ, TaPDIL1Aδ, and TaPDIL1B. The putative signal peptide (underlined), active center CGHC motifs (shaded in black), N-glycosylation consensus asparagine (shaded in gray), conserved arginine (box with straight lines), and glutamic acid (box with dotted lines) are indicated. Asterisks indicate positions that have a single, fully conserved residue.
