# Supplementary material for: Expression and characterization of protein disulfide isomerase family proteins in bread wheat
Source: BMC Plant Biol. 2015 Mar 4;15:73. doi: 10.1186/s12870-015-0460-2 (PMC4355359; doi:10.1186/s12870-015-0460-2)
Supplement: Additional file 2: Figure S2. — Alignment of amino acid sequences of TaPDIL1Aα and soybean GmPDIL-1. [file 12870_2015_460_MOESM2_ESM.pdf]

```
TmPDIL1Aα  26 EEAAAAEEAA AAPEAVLTLH ADNFDDAIAK HPFILVEFYA PWCGHCKSLA PEYEKAAQLL 85
GmPDIL-1   38 -------EES SEKEFVLTLD HSNFHDTVSK HDFIVVEFYA PWCGHCKKLA PEYEKAASIL 90
                     * : :  * ****.  .**.*:::* * **:***** *******.** *******.:*

TmPDIL1Aα  86 SKHDPAIVLA KVDANDEKNK PLAGKYEVQG FPTLKIFRNG GKNIQEYKGP REAEGIVEYL 145
GmPDIL-1   91 SSHDPPVVLA KIDANEEKNK DLASQYDVRG YPTIKILRNG GKNVQEYKGP READGIVDYL 150
              *.***.:*** *:***:****  **.:*:*:* :**:**:*** ***:****** ***:***:**

TmPDIL1Aα 146 KKQVGPASKE IKAPEDAT-Y LEDGKIHIVG VFTEFSGTEF TNFLELAEKL RSDYDFGHTV 204
GmPDIL1-1 151 KKQSGPASTE IKSADEATAF IGENKVAIVG VFPKFSGEEF DNFSALAEKL RSDYDFGHTL 210
              *** ****.* **:.::** : : :.*: *** **.:*** **  **  ***** *********:

TmPDIL1Aα 205 HANHLPRGDA AVERPLVRLF KPFDELVVDS KDFDVSALEK FIDASSTPKV VTFDKNPDNH 264
GmPDIL-1  211 NAKHLPRGES SVSGPVVRLF KPFDELFVDF QDFNVEALEK FVEESSTPVV TVFNNEPSNH 270
              :*:*****:: :*. *:**** ******.**  :**:*.**** *:: **** * ..*:::*.**

TmPDIL1Aα 265 PYLLKYFQSN APKAMLFLNF STGPFESFKS AYYGAVEEFS GKDVKFLIGD IEASQGAFQY 324
GmPDIL1-1 271 PFVVKFFNSP NAKAMLFINF TAEGAEAIKS KYREAAEQYK QQGVSFLVGD VESSQGAFQY 330
              *:::*:*:*   .*****:** ::   *::**  *  *.*::.  :.*.**:** :*:*******

TmPDIL1Aα 325 FGLKEDQAPL ILIQDSDSKK FLKEQVEAGQ IVAWLKDYFD GKLTPFRKSE PIPEANNEPV 384
GmPDIL-1  331 FGLKEEQVPL IIIQHNDGKK FFKPNLEADH IPTWLKAYKD GHVAPFVKSE PIPETNDEPV 390
              *****:*.** *:**..*.** *:* ::**.: * :*** * * *:::** *** ****:*:***

TmPDIL1Aα 385 KVVVADNIHD VVFKSGKNVL IEFYAPWCGH CKKLAPILDE AAATLQSEED VVIAKIDATA 444
GmPDIL-1  391 KVVVGASLED IVFKSGKNVL LEFYAPWCGH CKQLAPILDE VAISYQNEAD VVIAKLDATA 450
              ****. .:.* :********* :********* **:******* .* : *.* * *****:****

TmPDIL1Aα 445 NDVPGE-FDV QGYPTLYFVT PSGKKVSYEG GRTADEIVDY IKKNKETAGQ AAAAATEKAA 503
GmPDIL-1  451 NDIPSETFDV QGYPTVYFRS ASGKLSQYDG GRTKEDIIEF IEKNRDKPAQ QEQGKDEQEQ 510
              **:*.* *** *****:** : .***  .*:* *** ::*::: *:**::...*    .  *:

TmPDIL1Aα 504 EPAATEPLKDEL 515
GmPDIL-1  511 G-------KDEL 515
                      ****
```

Figure S2. Alignment of the amino acid sequences of TaPDIL1Aα and soybean GmPDIL-1. The active center CGHC motifs (shaded in black), conserved arginine (box with straight lines), and glutamic acid (boxes with dotted lines) are indicated. Asterisks indicate the positions that have a single, fully conserved residue. Colon indicates conservation between groups of strongly similar properties. Period indicates conservation between groups of weakly similar properties.
