# Supplementary material for: Expression and characterization of protein disulfide isomerase family proteins in bread wheat
Source: BMC Plant Biol. 2015 Mar 4;15:73. doi: 10.1186/s12870-015-0460-2 (PMC4355359; doi:10.1186/s12870-015-0460-2)
Supplement: Additional file 3: Figure S3. — Alignment of amino acid sequences of TaPDIL2 and TaPDIL2-1. [file 12870_2015_460_MOESM3_ESM.pdf]

```
TaPDIL2        1 MAAMPMPRSL LLILLLATPL LILPLAAAAV PTSNPDIDLE YLIKNAGLDD PTPATTATDP  60
TaPDIL2-1      1 MAAMPMPRSL LLILLLATPL LILPLAAAAV PTSNPDIDLE YLIKNAGLDD PTPATTATDP  60
                 ********** ********** ********** ********** ********** **********

TaPDIL2       61 EDDGAPDFPG LDADYDDEDL FGDDDGPEED SSHPSAADEA HVLLLTAANF TSVLAARRHV 120
TaPDIL2-1     61 EDDGAPDFPG LDADYDDEDL FGDDDGPEED SSHPSAADEA HVLLLTAANF TSVLAARRHV 120
                 ********** ********** ********** ********** ********** **********

TaPDIL2      121 MVEFYAPWCG HCRALAPHYA AAASALAEQG VDVALAKVDA TEDHDLAQAH GVQGYPTLLF 180
TaPDIL2-1    121 MVEFYAPWCG HCRALAPHYA AAASALAEQG VDVALAKVDA TEDHDLAQAH GVQGYPTLLF 180
                 ********** ********** ********** ********** ********** **********

TaPDIL2      181 FIDGVPRDYA GERTKDAIVA WISKKLGPAV QNLTTADEAE KIVTGDDVAV LAYLDHLSGA 240
TaPDIL2-1    181 FIDGVPRDYA GERTKDAIVA WISKKLGPAV QNLTTADEAE KIVTGDDVAV LAYLDHLSGA 240
                 ********** ********** ********** ********** ********** **********

TaPDIL2      241 HSDELAAASR LEDTISFYQT TSPDVAKLFH IDPEAKRPSV VLLKKEEEKL TVFDGEFRAS 300
TaPDIL2-1    241 HSDELAAASR LEDTISFYQT TSPDVAKLFH IDPEAKRPSV VLLKKEEEKL TVFDGEFRAS 300
                 ********** ********** ********** ********** ********** **********

TaPDIL2      301 AIAEFVSANK IPLITTLTQE TAPAIFDNPI KKQILLFAVA KESPQFLPII KETAKSFKGK 360
TaPDIL2-1    301 AIAEFVSANK IPLITTLTQE TAPAIFDNPI KKQILLFAVA KGSPQFLPII KETAKSFKGK 360
                 ********** ********** ********** ********** * ******** **********

TaPDIL2      361 LLFVFVERDN EEVGEPVANY FGIAGQETTV LAYTGNEDAK KFFFSGEISL DTIKEFAQDF 420
TaPDIL2-1    361 LLFVFVERDN EEVGEPVANY FGIAGQETTV LAYTGNEDAK KFFFSGEISL DTIKEFAQDF 420
                 ********** ********** ********** ********** ********** **********

TaPDIL2      421 LEDKLTPSYK SDPVPESNDE DVKVVVGKSL DQIVLDESKD VLLEVYAPWC GHCQSLEPIY 480
TaPDIL2-1    421 LEDKLTPSYK SDPVPESNDE DVKVVVGKSL DQIVLDESKD VLLEVYAPWC GHCQSLEPIY 480
                 ********** ********** ********** ********** ********** **********

TaPDIL2      481 NKLAKYLRGI DSLVIAKMDG TNNEHPRAKP DGFPTILFYP AGKKSFEPIT FEGDRTVVEM 540
TaPDIL2-1    481 NKLAKYLRGI DSLVIAKMDG TNNEHPRAKP DGFPTILFYP AGKKSFEPIT FEGDRTVVEM 540
                 ********** ********** ********** ********** ********** **********

TaPDIL2      541 YKFLKKHAAI PFKLKRPDSS AARTD---GP GSTAEGEKSS GSNPKDEL 585
TaPDIL2-1    541 YKFLKKHAAI PFKLKRPDSS AARTDGADGS GSTTEGEKSS GSNPKDEL 588
                 ********** ********** *****   *  *** ****** ********
```

**Figure S3. Alignment of the amino acid sequences of TaPDIL2 and TaPDIL2-1*.** The putative signal peptide (underlined), active center CGHC motifs (shaded in black), N-glycosylation consensus asparagine (shaded in gray), conserved arginine (box with straight lines), and glutamic acid (boxes with dotted lines) are indicated. *GenBank accession number: AK07151
