# Supplementary material for: Expression and characterization of protein disulfide isomerase family proteins in bread wheat
Source: BMC Plant Biol. 2015 Mar 4;15:73. doi: 10.1186/s12870-015-0460-2 (PMC4355359; doi:10.1186/s12870-015-0460-2)
Supplement: Additional file 4: Figure S4. — Alignment of amino acid sequences of TaPDIL2 and soybean GmPDIL-2. [file 12870_2015_460_MOESM4_ESM.pdf]

```
TaPDIL2     29 AVPTSNPDID LEYLIKNAGL DDPTPATTAT DPEDDGAPDF PGLDADYDDE DLFGDDDGPE  88
GmPDIL-2    26 --LTDDEDLG F--------L DEPS------ -----AAPEH G---HYHDDD ANFGDFEEDP  61
                  *.: *:. :        * *:*:            .**:.       :**:   *** :

TaPDIL2     89 EDSSHPSAAD EAHVLLLTAA NFTSVLAARR HVMVEFYAPW CGHCRALAPH YAAAASALAE 148
GmPDIL-2    62 EAYKQP-EVD EKDVVILKEK NFTDTVKSNR FVMVEFYAPW CGHCQALAPE YAAAATELKG 120
               *  .:*  .* * .*::*.   ***..: :.* .********* ****:****. *****: *

TaPDIL2    149 QGVDVALAKV DATEDHDLAQ AHGVQGYPTL LFFIDGVPRD YAGERTKDAI VAWISKKLGP 208
GmPDIL-2   121 E--DVILAKV DATEENELAQ QYDVQGFPTV YFFVDGIHKP YNGQRTKDAI MTWIKKKIGP 178
               :  ** **** ****:::***  :.***:**:  **:**: :  * *:****** ::**.**:**

TaPDIL2    209 AVQNLTTADE AEKIVTGDDV AVLAYLDHLS GAHSDELAAA SRLEDTISFY QTTSPDVAKL 268
GmPDIL-2   179 GIYNLTTVED AQRILTNETK VVLGFLNSLV GPESEELAAA SRLEDDVNFY QTVDPDVAKL 238
               .: ****.:: *::*:*.:.   **.:*: *  *..*:***** ***** :.** **..******

TaPDIL2    269 FHIDPEAKRP SVVLLKKEEE KLTVFDGEFR ASAIAEFVSA NKIPLITTLT QETAPAIFDN 328
GmPDIL-2   239 FHIDPDVKRP ALILVKKEEE KLNHFDGKFE KSEIADFVFS NKLPLVTIFT RESAPSVFEN 298
               *****:.*** :::*:***** **. ***:*.  * **:** : **:**:* :* :*:**::*:*

TaPDIL2    329 PIKKQILLFA VAKESPQFLP IIKETAKSFK GKLLFVFVER DNEEVGEPVA NYFGIAGQET 388
GmPDIL-2   299 PIKKQLLLFA TSNDSEKLIP AFKEAAKSFK GKLIFVYVEM DNEDVGKPVS EYFGISGNAP 358
               *****:**** .:::* :::*  :**:***** ***:**:**  ***:**:**: :****:*: .

TaPDIL2    389 TVLAYTGNED AKKFFFSGEI SLDTIKEFAQ DFLEDKLTPS YKSDPVPESND EDVKVVVGK 448
GmPDIL-2   359 KVLGYTGNDD GKKFVLDGEV TADKIKAFGD DFLEDKLKPF YKSDPVPESND GDVKIVVGN 418
               .**.****:* .***.:.**: : *.** *.: *******.*  ***********  ***:***:

TaPDIL2    449 SLDQIVLDES KDVLLEVYAP WCGHCQSLEP IYNKLAKYLR GIDSLVIAKM DGTNNEHPRA 508
GmPDIL-2   419 NFDEIVLDES KDVLLEIYAP WCGHCQALEP IYDKLAKHLR NIESLVIAKM DGTTNEHPRA 478
               .:*:****** ******:*** ******:*** **:****:** .*:******* ***.******

TaPDIL2    509 KPDGFPTILF YPAGKKSFEP ITFEGDRTVV EMYKFLKKHA AIPFKLKRPD SSAARTDGPG 568
GmPDIL-2   479 KPDGFPTLLF FPAGNKSFDP ITVDTDRTVV AFYKFLKKHA SIPFKLQKPT S---TSDAKG 535
               *******:** :***:***:* **.: *****  :******** :*****::*  *    :*. *

TaPDIL2    569 STAEGEKSSG SNPKDEL 585
GmPDIL-2   536 SSDAKE-SQS SDVKDEL 551
               *:   * *.. *: ****
```

Figure S4. **Alignment of the amino acid sequences of TaPDIL2 and soybean GmPDIL-2.** The active center CGHC motifs (shaded in black), conserved arginines (box with straight lines), and glutamic acid (boxes with dotted lines) are indicated. Asterisks indicate positions that have a single, fully conserved residue. Colon indicates conservation between groups of strongly similar properties. Period indicates conservation between groups of weakly similar properties.
