# Supplementary material for: Expression and characterization of protein disulfide isomerase family proteins in bread wheat
Source: BMC Plant Biol. 2015 Mar 4;15:73. doi: 10.1186/s12870-015-0460-2 (PMC4355359; doi:10.1186/s12870-015-0460-2)
Supplement: Additional file 6: Figure S6. — Alignment of amino acid sequences of TaPDIL4D, soybean GmPDIS-1, and soybean GmPDIS-2. [file 12870_2015_460_MOESM6_ESM.pdf]

```
TaPDIL4D   31 DEVLALTEST FEKEVGQDRG ALVEFYAPWC GHCKKLAPEY EKLAASFKKA KSVLIAKVDC 90
GmPDIS-1   28 DDVVVLSEDN FEKEVGQDRG ALVEFYAPWC GHCKKLAPEY EKLGSSFKKA KSVLIGKVDC 87
GmPDIS-2   27 DDVVALTEET FENEVGKDRA ALVEFYAPWC GHCKRLAPEY EQLGASFKKT KSVLIAKVDC 86
              *:*:.*:*.. ** *** *** ********** ****:***** * *.:****  *****.****

TaPDIL4D   91 DEHKSVCSKY GVSGYPTIQW FPKGSLEPKK YEGQRTAEAL TEYVNSEAAT NVKIAAVPSS 150
GmPDIS-1   88 DEHKSLCSKY GVSGYPTIQW FPKGSLEPKK YEGPRTAESL VEFVNTEGGT NVKIATVPSN 147
GmPDIS-2   87 DEHKSVCGKY GVSGYPTIQW FPKGSLEPKK YEGARTAEAL AAFVNIEAGT NVKIASVASS 146
              *****:* ** ********** ********** *** ****:* . :** *..* *****:* *.

TaPDIL4-1 151 VVVLTEETFD SVVLDETKDV LVEFYAPWCG HCKSLAPIYE KVASVFKQDE GVVIANLDAD 210
GmPDIS-1  148 VVVLTPENFN EVVLDETKDV LVEFYAPWCG HCKSLAPTYE KVATAFKLEE DVVIANLDAD 207
GmPDIS-2  147 VVVLSPNNFD EVVFDETKDV LVEFYAPWCG HCKALAPIYE KVAAAFNLDK DVVIANVDAD 206
              ****: :.*: .**:****** ********** ***.*** ** ***:.*. :  .*****:***

TaPDIL4D  211 KYTSLAEKYG VSGFPTLKFF PKGNKAGEEY ESGRELDDFV KFINEKSGTS RDSKGQLTSE 270
GmPDIS-1  208 KYRDLAEKYD VSGFPTLKFF PKGNKAGEDY GGGRDLDDFV AFINEKSGAS RDGKGQLTSQ 267
GmPDIS-2  207 KYKDLAEKYG VSGYPTLKFF PKSNKAGENY DGGRDLDDFV AFINEKCGTY RDGKGQLTSK 266
              ** .*****. ***.****** **.*****:*  .**:*****  *****.*:  **.******

TaPDIL4D  271 AGLVASLDAL VKEFHSAADD KRKEILSKIE EEAAKLSGPA VKHGKIYVNV AKKILQKGSD 330
GmPDIS-1  268 AGIVESLDVL VKEFVAASDE EKKSVFTRLE EEVVKLKGSA SRYGKIYLKA AKNYREKGSD 327
GmPDIS-2  267 AGIIASLDDL VKEFVSADSN EKKAVYSRLE EEVKKLKGSS ARHGDLYLKL AKKGMEKGAD 326
              **:. ***.* **** :*: : ::*.:.:::* **..**.*.. ::** :*:.  *:  ::**.*

TaPDIL4D  331 YTKKETERLH RLLEKSISPS KADEFAIKKN ILSAFSS 367
GmPDIS-1  328 YAKNEIQRLQ RILDKSISPA KADELTLKKN ILSTYAA 364
GmPDIS-2  327 YAKNEIQRLE RMLEKSVSPA KADEFTLKKN ILSIFA  362
              *:*:* :**  *.*:**:**: ****:::*** ***:::
```

Figure S6. Alignment of the amino acid sequences of TaPDIL4D, soybean GmPDIS-1, and soybean GmPDIS-2. The active center CGHC motifs (shaded in black), conserve arginine (box with straight lines), and glutamic acid (boxes with dotted lines) are indicated. Asterisks indicate positions that have a single, fully conserved residue. Colon indicates conservation between groups of strongly similar properties. Period indicates conservation between groups of weakly similar properties.
