# Supplementary material for: Expression and characterization of protein disulfide isomerase family proteins in bread wheat
Source: BMC Plant Biol. 2015 Mar 4;15:73. doi: 10.1186/s12870-015-0460-2 (PMC4355359; doi:10.1186/s12870-015-0460-2)
Supplement: Additional file 7: Figure S7. — Alignment of amino acid sequences of TaPDIL5A and soybean GmPDIM. [file 12870_2015_460_MOESM7_ESM.pdf]

```
TaPDIL5A    23 LYSAGSPVHQ LNPNNFK-KV LNANGVVLVE FFAPWCGHCK QLTPIWEKAA GVLKGVATVA 81
αGmPDIM     29 LYGASSPVLQ LTPSNFKSKV LNSNGVVLVE FFAPWCGHCQ ALTPIWEKAA TVLKGVVTVA 88
               **.*.*** * *.*.*** ** **:******* *********:  *********  *****.***

TaPDIL5A    83 ALDADAHKEL AQQYGIRGFP TIKVFLPGKP PVDYEGARDV KPIVNFALSQ VKGLLRDRLD 141
GmPDIM      89 AIDADAHPSL AQEYGIRGFP TIKVFAPGKP PVDYQGARDA KPIAEFALQQ VKALLKDRLS 148
               *:***** .* **:******* ***** **** ****:****. ***.:***.* **.**:***.

TaPDIL5A   142 GKTSGGSSGK TSGGSSEKKN EPNESVELNS SNFDELVVKS KDLWIVEFFA PWCGHCKKLA 201
GmPDIM     149 GKATGGSSDK T--------- ETSSSVELNS GNFDELVIKS KELWIVEFFA PWCGHCKKLA 199
               **::****.* *          *...****** .******:** *:******** **********

TaPDIL5A   202 PEWKRAAKNL KGQVKLGHVD CDSDKSLMSK YKVEGFPTIL VFGADKESPF PYQGARAASA 261
GmPDIM     200 PEWKKASNSL KGKVKLGHVD CDAEKSLMSR FKVQGFPTIL VFGADKDSPI PYEGARTALA 259
               ****:*::.* **:******* **::*****: :**:****** ******:**: **:***:* *

TaPDIL5A   262 IEPFALEQLE ANAAPPEVSE LTSADVMEEK CASAAICFVS FLPDILDSKA EGRNKYLELL 321
GmPDIM     260 IESFALEQLE TNVAPPEVTE LHSPDVLEEK CGSAAICFVA FLPDILDSKA EGRNIYLQQL 319
               **.******* :*.*****:* * *.**:*** *.*******: ********** **** **: *

TaPDIL5A   322 LSVAEKFKKS PYSFVWAGAG KQADLEKQVG VGGYGYPAMV ALNVKKGAYA PLRSAFELAE 381
GmPDIM     320 LSVAEKFKRS PYSYVWVAAG NQPDLEKNVG VGGYGYPALV ALNLKKAVYA PLKSAFELDQ 379
               ********:* ***:**..** :*.****:** ********:* ***:**..** **:***** :

TaPDIL5A   382 ITEFVKEAGR GGKGNLPLEG APTVVESEPW DGKDGEVIEE DEFSLEELMA DSSAPNDEL 440
GmPDIM     380 IIEFVKEAGR GGKGNLPLQG TPTIVKTEPW DGKDGEIIEE DEFSLEELMG EDASSKDEL 439
               * ******** ********:* :**:*::*** ******:*** *********. :.::.:***
```

Figure S7. Alignment of amino acid sequences of TaPDIL5A and soybean GmPDIM. The active center CGHC motifs (shaded in black), conserved arginine (box with straight lines), and glutamic acid (boxes with dotted lines) are indicated. Asterisks indicate positions that have a single, fully conserved residue. Colon indicates conservation between groups of strongly similar properties. Period indicates conservation between groups of weakly similar properties.
