# Supplementary material for: Expression and characterization of protein disulfide isomerase family proteins in bread wheat
Source: BMC Plant Biol. 2015 Mar 4;15:73. doi: 10.1186/s12870-015-0460-2 (PMC4355359; doi:10.1186/s12870-015-0460-2)
Supplement: Additional file 8: Figure S8. — Caryopsis weight after anthesis. [file 12870_2015_460_MOESM8_ESM.pdf]

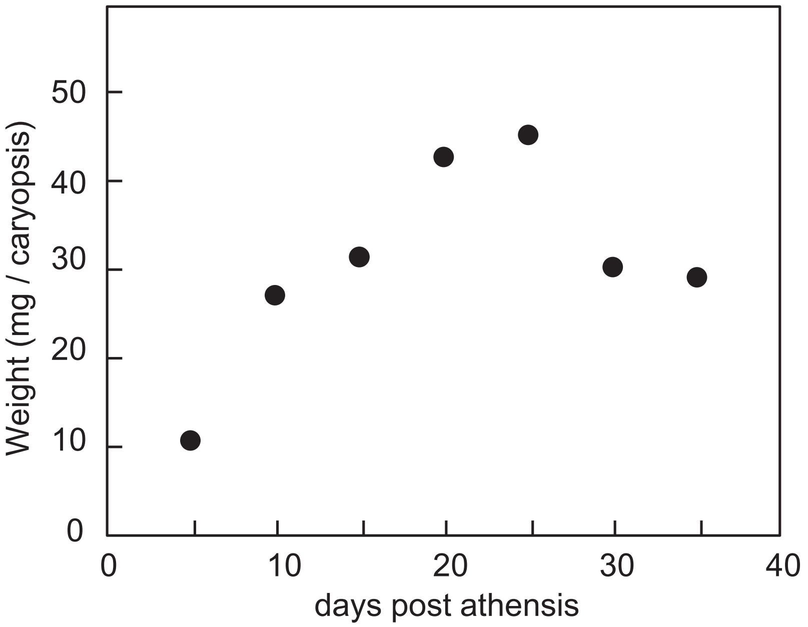

Figure S8. Caryopsis weight after anthesis. Wheat caryopses (30–100 grains) were collected and weighed. Average values per caryopsis were plotted.
