# Supplementary material for: Expression and characterization of protein disulfide isomerase family proteins in bread wheat
Source: BMC Plant Biol. 2015 Mar 4;15:73. doi: 10.1186/s12870-015-0460-2 (PMC4355359; doi:10.1186/s12870-015-0460-2)
Supplement: Additional file 9: Figure S9. — Dot blot analysis of recombinant wheat PDI family proteins. [file 12870_2015_460_MOESM9_ESM.pdf]

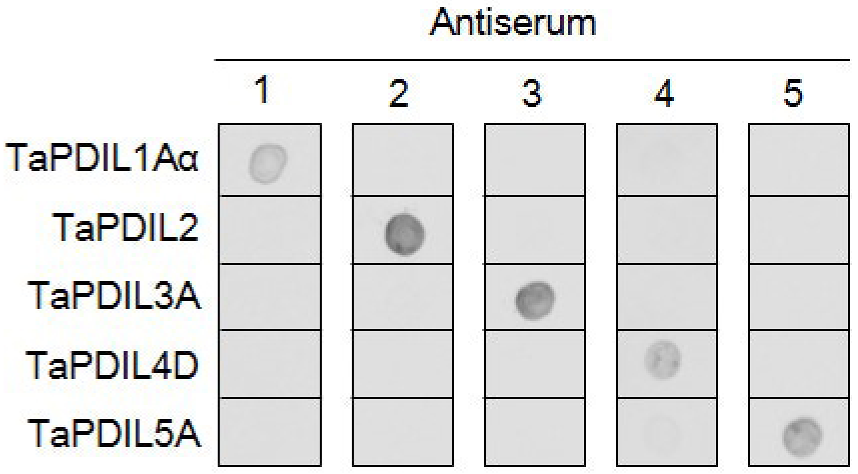


**Figure S9. Dot blot analysis of recombinant wheat PDI family proteins.** Recombinant TaPDIL1Aα, TaBPDIL2, TaPDIL3A, TaPDIL4D, and TaPDIL5A (each 10 ng/5 µL) were spotted on a nitrocellulose membrane and immunolabeled with anti-TaPDIL1Aα (1), anti-TaPDIL2 (2), anti-TaPDIL3A (3), anti-TaPDIL4D (4), or anti-TaPDIL5A serum (5).
