# Supplementary material for: Expression and characterization of protein disulfide isomerase family proteins in bread wheat
Source: BMC Plant Biol. 2015 Mar 4;15:73. doi: 10.1186/s12870-015-0460-2 (PMC4355359; doi:10.1186/s12870-015-0460-2)
Supplement: Additional file 5: Figure S5. — Alignment of amino acid sequences of TaPDIL3A and soybean GmPDIL-3a. [file 12870_2015_460_MOESM5_ESM.pdf]

```
TaPDIL3A   24 AKLDLDEVDD SEVLEALLAV DEEEEDAAPP GGG--GGAEA VRRTQSMVLV LDNDNAARAV  81
GmPDIL3a   25 ------EVKD -E-LEELLAV DEEVEREAEK GGEKLSEAEV LSKAQRIVIE LKNENTERVV  76
                    **.*  * ** **** *** *  **  **.  . **. : ::* :*:  *.*:*: *.*

TaPDIL3A   82 RDHPELLLLG YAPWCERSAK LMPRFAEAAA ALRAMGSAVA FAKLDGERFP KAASTVGVNG 141
GmPDIL3a   77 NGNEFVLVLG YAPWCPRSAE LMPHFAEAAT SLKELGSPLI MAKLDADRYP KPASFLGVKG 136
              ..:  :*:** ***** ***: ***:*****: :*: :**.:  :****.:*:* *.** :**:*

TaPDIL3A  142 FPSVLLFVNG TEHAYTGLHT KDAIVTWVRK KTGTPVIRIE SKDSAEELLK KGQTFALGLF 201
GmPDIL3a  137 FPTLLLFVNG TSQPYSGGFT ADDIVIWAQK KTSTPVIRIS SVAEAEKFLT KYQTFLIGRF 196
              **::****** *.:.*:* .*  * ** *.:* **.******. *  .**::*. * *** :* *

TaPDIL3A  202 KNYEGTDHEE FMKAATAENE VQFVETNDRN VAKILFPGIA SEEQFLGLVK SEPEKFEKFD 261
GmPDIL3a  197 ENFEGPDYEE FVSAAKSDNE IQFVETSQVE LAQVLYPDIK PTDRFLGIVK SEPERYSAYD 256
              :*:**.*:** *:.**.::** :*****.: : :*::*:*.*  . ::***:** ****::. :*

TaPDIL3A  262 GAFEEKEILQ FVELNKFPLI TVFTDLNSAK VYSSPIKLQV FTFAEAYDFE DLESIVQEVA 321
GmPDIL3a  257 GAFILNKILE FVDYNKFPLV TKLTEMNSVR VYSSPIKLQV LVFANIDDFK NLLDTLQDVA 316
              ***  ::**: **: *****: * :*::**.: ********** :.**:  **: :* . :*:**

TaPDIL3A  322 RGFKTKIMFI YVDTAEENLA KPFLTLYGLE GDKPTVT-AF DTSKGAKYVL EADINAKNLK 380
GmPDIL-3a 317 KTFKSKIMFI YVDINDENLA KPFLTLFGLE ESKNTVVGAF DNSMSSKYLL ESKPTQINIE 376
              : **:***** ***  :**** ******:***  .* **. ** *.* .:**:* *:. .  *::

TaPDIL3A  381 EFSLSLLDGT LPPYFRSEPV PQ-EEGLVEK VVGRTFDSSV LQSPHNILLE AHAPWCVDCE 439
GmPDIL-3a 377 EFCNNLMQGS LSPYFKSQPI PDNTEASVRA IVGKTFDDEI LSSKKDVLLE VFTPWCMNCE 436
              **. .*::*: *.***:*:*: *:  *. *.  :**:***..: *.* :::*** ..:***::**

TaPDIL3A  440 AISKNIEKLA KHFSGLDNLK FARIDASVNE HPKLQVNNYP TLLLYPAEDK TNPIKLSKKL 499
GmPDIL-3a 437 ATSKQVEKLA KHYKGSSNLI FARTDASANE HPKLQVNDYP TLLFYRADDK ANPIKLSTKS 496
              * **::**** **:.* .**  *** ***.** *******:** ***:* *:** :******.*

TaPDIL3A  500 SLKDMARFLK EKLQISDVEI KEKLQTPNIE TVAAADNVKD EL 541
GmPDIL-3a 497 SLKELAASIN KYLKVKNQVL ---------- --------KD EL 520
              ***::*  :: : *::.:  :                    ** **
```

Figure S 5. Figure S5. Alignment of amino acid sequences of TaPDIL3A and soybean GmPDIL-3a. The active center CXXC/S motifs (shaded in black), conserved glutamic acid (boxes with dotted lines) is indicated. Asterisk indicates positions which have a single, fully conserved residue. Colon indicates conservation between groups of strongly similar properties. Period indicates conservation between groups of weakly similar properties.
